# Corrosion as the origin of limited lifetime of vanadium oxide-based aqueous zinc ion batteries

**DOI:** 10.1038/s41467-022-29987-x

**Published:** 2022-05-02

**Authors:** Yangmoon Kim, Youngbin Park, Minkwan Kim, Jimin Lee, Ki Jae Kim, Jang Wook Choi

**Affiliations:** 1 School of Chemical and Biological Engineering and Institute of Chemical Processes, Seoul National University, 1 Gwanak-ro, Gwanak-gu, Seoul 08826 Republic of Korea; 2 Department of Energy Engineering, Konkuk University, Neungdong-ro 120, Gwangjin-gu, Seoul 05029 Republic of Korea

**Keywords:** Batteries, Batteries

## Abstract

Aqueous zinc ion batteries are receiving increasing attention for large-scale energy storage systems owing to their attractive features with respect to safety, cost, and scalability. Although vanadium oxides with various compositions have been demonstrated to store zinc ions reversibly, their limited cyclability especially at low current densities and their poor calendar life impede their widespread practical adoption. Herein, we reveal that the electrochemically inactive zinc pyrovanadate (ZVO) phase formed on the cathode surface is the main cause of the limited sustainability. Moreover, the formation of ZVO is closely related to the corrosion of the zinc metal counter electrode by perturbing the pH of the electrolyte. Thus, the dissolution of VO_2_(OH)_2_^−^, the source of the vanadium in the ZVO, is no longer prevented. The proposed amalgamated Zn anode improves the cyclability drastically by blocking the corrosion at the anode, verifying the importance of pH control and the interplay between both electrodes.

## Abstract (web summary)

Aqueous zinc ion batteries are good systems for large-scale energy storage. Here, the authors report that the corrosion of zinc metal anode is the origin of limited lifetime of vanadium oxide-based aqueous zinc ion batteries, and supressing corrosion improves the calendar and cycle lifetime markedly.

## Introduction

Taking advantage of their high energy density, long cyclability, and scalability in manufacturing, lithium-ion batteries (LIBs) have witnessed unprecedented success in powering a variety of applications ranging from portable electronic devices to electric vehicles and even as large as grid-scale energy storage systems. However, the high cost associated with the raw materials and safety concerns originating from the use of flammable carbonate electrolytes make it invaluable to explore alternative battery systems, particularly with the view of targeting large-scale applications. Along this direction, rechargeable aqueous batteries have been considered promising candidates owing to the various advantages stemming from the use of water: non-flammability, environmental benignity, high ionic conductivity of the electrolyte, and low manufacturing cost because drying facilities are superfluous. Among a number of aqueous rechargeable batteries, rechargeable aqueous zinc-ion batteries (AZIBs) stand out because the high theoretical capacity of the metallic Zn anode (5854 mAh cm^−3^) can largely overcome the inferior energy density resulting from the limited potential windows of aqueous media^1^. The additional advantages of metallic Zn are its low cost, natural abundance, high stability in an aqueous environment, and comparatively low redox potential (−0.76 V vs. the standard hydrogen electrode (SHE)).

In spite of the aforementioned advantages, identifying cathode materials that can accommodate divalent Zn ions and thus enable a reliable full-cell to be built is not trivial. The limited electrochemical window of an aqueous electrolyte (thermodynamically 1.23 V) and the sluggish diffusion of divalent Zn ions in a crystal lattice are the main hurdles in the way of discovering appropriate cathode materials^2^. Recently, various groups of materials including oxides^3–7^, sulfides^8–10^, metal-organic frameworks (MOFs)^11,12^, and organic molecules^13–15^ have been studied as cathode materials for AZIBs. Across the oxide and sulfide families, vanadium-containing materials^16–28^ have been intensively studied because the wide redox range of vanadium (V^5+^ ↔ V^4+^ ↔ V^3+^) increases its chances to fit in the limited potential window of water. Layered vanadium oxides (layer-VOs) are particularly promising because they offer high specific capacities based on the large interlayer distances that promote facile Zn^2+^ (de)intercalation.

Ever since the system of Zn_0.25_V_2_O_5_⋅nH_2_O in 1 M ZnSO_4(*aq*)_ was reported by Nazar’s group^29^, a series of layer-VOs with various structures and morphologies have been reported^16–25^. Nevertheless, layer-VOs still experience capacity fading over cycling especially when cycled slowly (i.e., <0.5 C). Many researchers have revealed that repeated electrochemical reactions cause the structural degradation of layer-VOs, resulting in dissolution and inactivation of the active phase. Based on this rationale, a considerable number of strategies utilizing the so-called “pillar effect” have been reported, where guest species such as cations (Li^+^, K^+^, NH_4_^+^, Mg^2+^, Ca^2+^, Ba^2+^, La^3+^, etc.)^30–37^ and polymers (polyaniline, PEDOT, etc.)^38,39^ were introduced between the layers of layer-VOs to sustain the framework. Although this pillar strategy made a substantial contribution to enhancing the cyclability of the layer-VOs, the observed performance remains unsatisfactory in terms of practical standards. The incorporation of conductive carbonaceous components^40^ and the use of a surface coating^41,42^ have also been adopted to address the structural degradation of layer-VOs, but have resulted in limited success. More frustratingly, vanadium-based materials beyond the layer-VO family are commonly characterized by structural instability and limited cyclability in AZIBs especially at low current densities, prompting us to reevaluate the fading mechanisms established thus far.

Here, using an aqueous electrolyte based on zinc trifluoromethanesulfonate (Zn(OTf)_2_) as an optimized electrolyte, we elucidate the degradation mechanism of layer-VO-based AZIBs by conducting a series of experiments in a systematic fashion. The carefully designed experiments inform that the capacity fading of layer-VOs is closely related to the formation of the zinc pyrovanadate (ZVO) phase on the electrode surface, and the formation of this phase is a combined outcome of the dissolution of vanadium from the cathode and the corrosion of the Zn metal anode. Based on this lesson, a corrosion-resistant metallic Zn anode was incorporated in an AZIB. This markedly improved the cyclability, suggesting a reason for the limited success of existing strategies to employ cathodes that are entirely vanadium-based and their AZIBs. This comprehensive investigation, which considers the entire cell as a system, offers a critical, yet useful viewpoint when a vanadium-based cathode pairs with metallic Zn anode.

## Results and discussion

### Cathode characterization

**Fig. 1:**
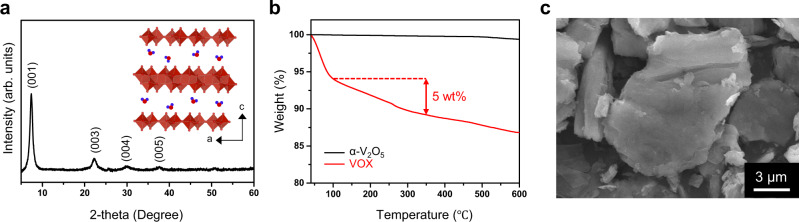
Characterization of as-prepared VOX. **a** XRD pattern of VOX. The inset is the crystal structure of VOX viewed along the b-axis. **b** TGA results of VOX and α-V_2_O_5._ **c** SEM image of VOX particle.

Vanadium pentoxide (α-V_2_O_5_) is one of the most widely studied layer-VOs because of its facile synthesis, low cost, and high theoretical specific capacity of 589 mAh g^−1^ based on the two-electron reaction^43^. In AZIBs, however, α-V_2_O_5_ is known to require long activation cycles, making it incompatible with practical use^21^. Recently, Huang’s group reported that α-V_2_O_5_ undergoes a transformation from its original phase to hydrated vanadium pentoxide within the aqueous zinc electrolyte, and once transformed, it does not need the activation step anymore^44^. With these results in mind, we used a simple sol-gel method to synthesize the hydrated vanadium pentoxide (vanadium oxide xerogel, VOX) to obviate the need for the activation step and employed it as the cathode in an AZIB. Figure 1a presents the X-ray diffraction (XRD) pattern of the as-synthesized VOX, which is well indexed to that of the designated layered phase^45^. The (001) peak located at 2θ ~7.4° suggests that VOX has a large interlayer distance of 12 Å owing to the intercalated water molecules. The hydration of VOX can also be confirmed by thermogravimetric analysis (TGA), which generally informs the intercalated water content based on the weight loss in the temperature range of 100–350 °C (Fig. 1b)^17^. According to this analysis, the water content of the synthesized VOX was 5 wt%. The weight loss below 100 °C is attributed to physically adsorbed water owing to the hygroscopicity of VOX. The morphology of VOX was examined by scanning electronic microscopy (SEM). As shown in Fig. 1c and Supplementary Fig. 1a, VOX exhibits a three-dimensional but somewhat flat and irregular architecture with its particles being a few micrometers in size. Furthermore, elemental mapping with energy-dispersive spectrometry (EDS) indicates the uniform distribution of vanadium and oxygen in the VOX without the presence of any guest species (Supplementary Fig. 1b).

**Fig. 2:**
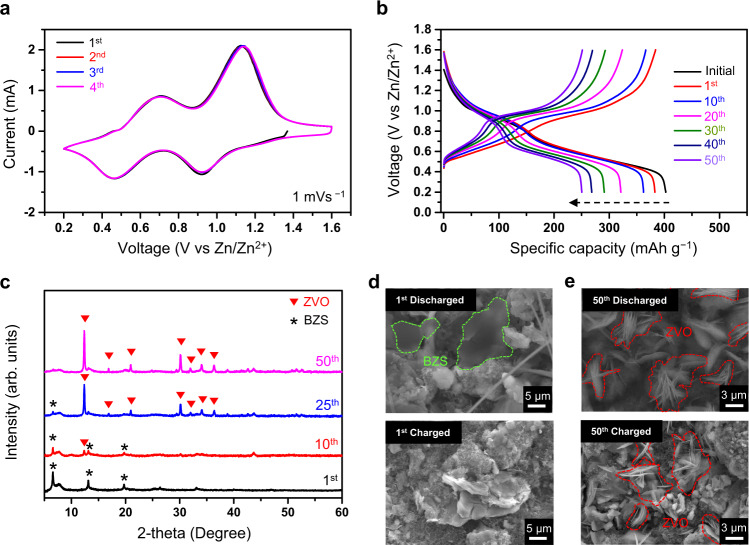
Electrochemical properties of VOX electrode and its structural and morphological analyses. **a** Cyclic voltammetry curves of VOX electrode at different cycle numbers when scanned at a scan rate of 1 mVs^−1^. **b** Galvanostatic charge-discharge profiles of VOX electrode at different cycle numbers when cycled at 200 mA g^−1^. **c** Ex-situ XRD patterns of discharged VOX electrode at different cycle numbers. **d**, **e** Ex-situ SEM images of VOX electrodes at different cycling points.

The electrochemical performance of the VOX electrode was evaluated in the typical coin-type cell configuration at room temperature, with the cell consisting of a VOX cathode, 3 m Zn(OTf)_2(*aq*)_ electrolyte, and a zinc metal anode. The trifluoromethanesulfonate (OTf^−^) anion was chosen because it offers lower Zn^2+^ desolvation energy based on its bulky size. When present in the electrical double layer on the electrode surface, this anion would be expected to limit side reactions involving water molecules owing to its hydrophobic nature^22,46^. Aside from the selection of the anion, the concentration of the electrolyte was selected to be 3 m (3 mol of Zn(OTf)_2_ in 1 kg of H_2_O) to take advantage of the so-called high concentration effect in the solubility range; the solubility of Zn(OTf)_2_ in water is ~4 m. Cyclic voltammetry tests that were performed at a scan rate of 1 mV s^−1^ showed that the curves maintained almost identical peak positions and intensity in the initial four cycles (Fig. 2a), which points to the fact that VOX was pre-activated. Figure 2b depicts the galvanostatic charge and discharge (GCD) curves of the VOX for the first 50 cycles at a current density of 200 mA g^−1^. The current density of 200 mA g^−1^ was taken because the degradation of layer-VOs in AZIBs is usually amplified at a moderately low current density (~0.3 C)^47^. The discharge capacity of 383 mAh g^−1^ that was delivered in the first cycle rapidly dropped to 250 mAh g^−1^ after 50 cycles (65.3% capacity retention).

### Degradation mechanism

At different cycling points, ex-situ XRD and SEM were employed to observe the structural and morphological changes in the degradation process. Figure 2c displays the ex-situ XRD patterns of the discharged VOX electrode at four different cycling points. Only broadened VOX peaks and three well-resolved peaks at 2θ ~6.58°, 13.1°, 19.68° were observed after the first cycle, and these three peaks correspond with the basic zinc salt (BZS) phase containing the OTf^−^ anion (Zn_12_(CF_3_SO_3_)_9_(OH)_15_·nH_2_O)^48^. As the number of cycles increases, the intensity of the BZS peaks decreases and instead new peaks appear. These new peaks at 2θ *~*12.38°, 16.86°, 20.96°, 30.14°, 31.96°, 34.02°, 36.34° are well indexed to the zinc pyrovanadate phase (ZVO, Zn_3_V_2_O_7_(OH)_2_·nH_2_O) (Supplementary Fig. 3). These peaks that were assigned to the ZVO phase were also observed for the degraded VOX electrode in the charged state (Supplementary Fig. 4), implying that the ZVO phase persists regardless of whether the electrode is in the charged or discharged state as cycling progresses. The identity of these by-products was also confirmed by acquiring ex-situ SEM images. Figure 2d shows the SEM images of the VOX electrode after the first discharge (top) and charge (bottom). By-products with a sheet-like morphology exist on the surface of the discharged electrode; however, they disappear after the subsequent charge. This is consistent with the result of the ex-situ XRD analysis of the selected state during the first cycle (Supplementary Fig. 2a) and is universally observed with the BZS produced in AZIBs^47,48^. After 50 cycles, by-products with similar sheet-like morphology were observed on the surface of the electrodes in both the charge and discharge states (Fig. 2e). This morphology differs slightly in appearance from that on the electrode after the first discharge. Moreover, at the 50^th^ cycle, the entire surface of the VOX electrode is covered with the by-products (Supplementary Fig. 5a, b). Additionally, the SEM-EDS analysis indicates that the by-products on the electrode surface after the 50^th^ charge do not include the sulfur originating from the OTf^−^ anion (Supplementary Fig. 5c, d). Rather, a larger amount of zinc was detected than vanadium even though zinc ions were extracted during charging. This seems to suggest that the by-products observed in the 50^th^ cycle must be ZVO rather than BZS. Furthermore, these series of results led us to suspect that the formation of the ZVO, which shows no electrochemical activity^49^, on the surface of the VOX electrode is strongly linked to the degradation of the cell performance.

Interestingly, the ex-situ XRD patterns (Supplementary Fig. 2a) did not exhibit any peaks related to the ZVO phase in any selected state in the first cycle, which implies that the ZVO phase did not form during the electrochemical charge /discharge process. Recently, the formation of the ZVO within the aqueous solution containing Zn ions was reported to be associated with the dissolution of vanadium pentoxide and the subsequent precipitation via the following reactions^44^.

1 $${{{{{\rm{Dissolution}}}}}}:{{{{{{\rm{V}}}}}}}_{2}{{{{{{\rm{O}}}}}}}_{5}+3{{{{{{\rm{H}}}}}}}_{2}{{{{{\rm{O}}}}}}\to 2\,{{{{{{\rm{VO}}}}}}}_{2}{{({{{{{\rm{OH}}}}}})}_{2}}^{-}+2\,{{{{{{\rm{H}}}}}}}^{+}$$

2 $${{{{{\rm{Precipitation}}}}}}: \,2\,{{{{{{\rm{VO}}}}}}}_{2}{{({{{{{\rm{OH}}}}}})}_{2}}^{-}+3\,{{{{{{\rm{Zn}}}}}}}^{2+}+3\,{{{{{{\rm{H}}}}}}}_{2}{{{{{\rm{O}}}}}}\\ \to {{{{{{\rm{Zn}}}}}}}_{3}{{{{{{\rm{V}}}}}}}_{2}{{{{{{\rm{O}}}}}}}_{7}{({{{{{\rm{OH}}}}}})}_{2}\cdot {{{{{{\rm{nH}}}}}}}_{2}{{{{{\rm{O}}}}}} \,({{{{{\rm{ZVO}}}}}})+4\,{{{{{{\rm{H}}}}}}}^{+}$$

To establish whether this also applies to the VOX, a simple dissolution test was performed by immersing the VOX electrode in a 3 m Zn(OTf)_2(*aq*)_ solution. The solution with the electrode was inspected every 24 h, yet the solution was not observed to have undergone a color change (Supplementary Fig. 6a), which is in contrast with the change that occurred when the VOX electrode was immersed in deionized water (Supplementary Fig. 6b). Nevertheless, when immersed in the 3 m Zn(OTf)_2(*aq*)_ solution for 120 h, intense peaks reflective of the ZVO phase were observed on the XRD pattern of the VOX electrode (Supplementary Fig. 6c). This observation of the formation of the ZVO phase is in good agreement with the dissolution-precipitation reactions presented above.

**Fig. 3:**
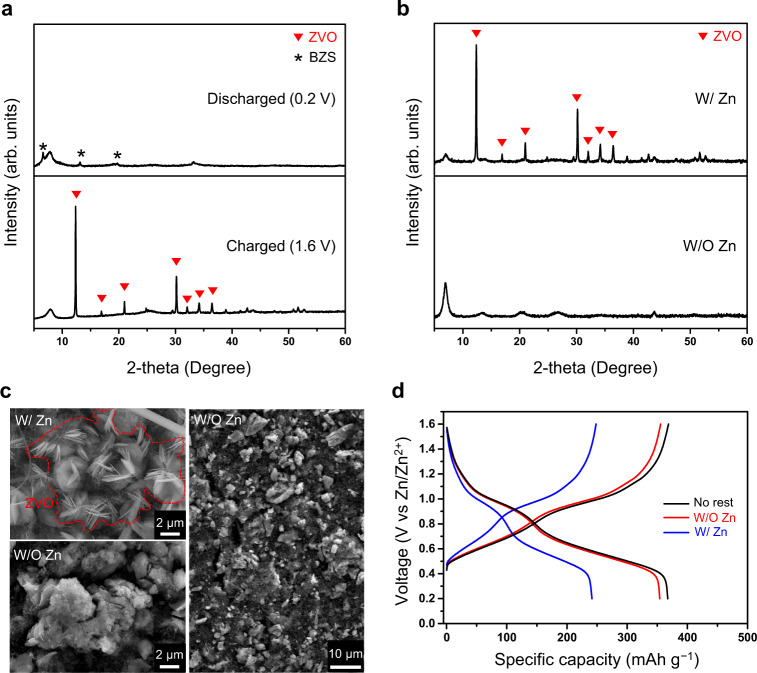
Dissolution test under various conditions. **a** Ex-situ XRD patterns of VOX electrodes after reaching different charge/discharge states followed by 5 days of rest. **b** Ex-situ XRD patterns of VOX electrodes after 5 days of rest at charged state with and without zinc metal counter electrode. **c** SEM images of VOX electrodes after 5 days of rest at charged state with and without zinc metal counter electrode. **d** The first galvanostatic charge and discharge profiles of VOX electrodes under different test conditions.

The conditions under which the dissolution test was carried out using an excessive amount of electrolyte are far from those of practical electrochemical cells. All of our electrochemical and dissolution tests were performed using coin-type cells containing the electrolyte, the amount of which was such that it barely wet the separator, to benchmark the conditions of commercial cells as much as possible (Supplementary Fig. 7a). In an effort to elucidate the formation of the ZVO phase, we first determined the effect of the charge/discharge state of the VOX on the degree of ZVO formation. To this end, two cells at different states of charge (SOCs) were prepared; the first was discharged to 0.2 V and the other was charged to 1.6 V. Subsequently, these two cells were maintained under rest for five days, after which they were subjected to XRD analysis. According to the XRD results, the peaks corresponding to the ZVO phase were detected only for the charged VOX electrode (Fig. 3a). The formation of the ZVO phase was also reflected in the OCV (open circuit voltage) change when the cell was subjected to rest for five days. It took the OCV of the charged VOX much longer to reach the equilibrium potential compared to its discharged counterpart (Supplementary Fig. 8) because the dissolution of vanadium oxide and subsequent precipitation of ZVO during the charge process disturb the cell from relaxing. Aside from these observations, the XPS results of V 2*p*_3/2_ (Supplementary Fig. 2b, c) show that the valence state of vanadium in the charged state is dominantly +5 whereas the oxidation states of +4 and +3 emerge at the expense of the +5 state in the discharged state. These combined results led us to build the rationale that, as the valence state of vanadium in VO_2_(OH)_2_^−^ in reaction (2) is +5, the precipitation mainly occurs during the charging process. In addition, the lattice was observed to contract by ~1 Å during the first discharge (Supplementary Fig. 2a), implying that the intercalated Zn ions screen the interlayer electrostatic repulsion^29^. This interpretation can also be understood to indicate that VOX is more robust against vanadium dissolution in the discharged state.

Remarkably, a significant amount of the ZVO phase still formed in the dissolution test in the coin-type cell even though the amount of electrolyte was decreased by more than 95% compared to the test in the vial (Supplementary Fig. 6a). Another major difference between the dissolution tests in the coin-cell and vial was the presence of zinc metal. Noting this major difference, we prepared two different coin cells; one in which the VOX electrode was paired with a zinc metal counter electrode (W/Zn) and the other without the counter electrode (W/O Zn). Based on the observation that the ZVO is produced on the charged VOX electrode, the VOX cells were subjected to the dissolution tests in the charged state. To prepare the W/O Zn cell, a cell was first charged and then re-assembled after removing the zinc metal electrode (Supplementary Fig. 7b). When analyzed using XRD and SEM after 5 days of rest, surprisingly, the peaks associated with the ZVO were not detected for the VOX electrode in the W/O Zn cell (Fig. 3b, bottom). In stark contrast, the W/Zn cell clearly exhibited the characteristic peaks of the ZVO phase (Fig. 3b, top). These XRD results are consistent with the SEM images (Fig. 3c). The morphologies ascribed to the ZVO phase were not observed for the W/O Zn cell at all, unlike its W/Zn cell counterpart. Additionally, the VOX electrodes that underwent the dissolution tests were subjected to GCD tests by replacing the electrolyte and zinc metal anode with fresh ones, respectively. After one cycle of activation, the GCD curves were obtained as shown in Fig. 3d. Compared to the cell that did not undergo the dissolution test (denoted as “no rest”), the VOX electrodes from the W/Zn and W/O Zn cells showed capacity loss of 36% and 4%, respectively. All in all, we reached the conclusion that the formation of the ZVO is a critical factor responsible for the degradation of the VOX electrode, and the presence of the Zn metal counter electrode plays an important role therein.

**Fig. 4:**
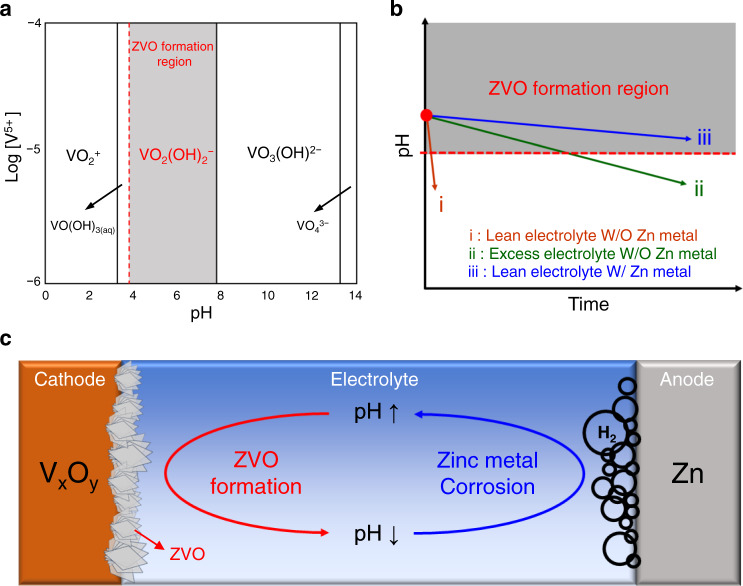
Dissolution of vanadium oxides and their degradation mechanism in full-cell system. **a** Predominant V^5+^ species in aqueous solutions at 25 °C as a function of vanadium concentration and pH^55^. **b** Schematic illustration of pH change rate of electrolyte according to test conditions and its correlation with the formation of ZVO. **c** Schematic illustration of chemical degradation loop in an AZIB cell containing layer-VO.

In an attempt to understand the formation of the ZVO phase more deeply, we paid attention to the diagram of V^5+^ at different concentrations and pH levels in aqueous media (Fig. 4a). According to this diagram, V^5+^ exists in the form of VO_2_(OH)_2_^−^ under mildly acidic and basic conditions (shaded area in Fig. 4a). On the other hand, Eqs. (1) and (2) indicate that a proton is produced in both the dissolution and precipitation reactions. Thus, as the ZVO is formed on the surface of the VOX electrode, the electrolyte becomes more acidic. With this acidification, the pH of the electrolyte could fall below the region in which VO_2_(OH)_2_^−^ exists, whereupon the formation of ZVO is no longer feasible. Recall that VO_2_(OH)_2_^−^ arises from the dissolution of vanadium from VOX and yields the ZVO phase upon reacting with Zn ions from the electrolyte^44^. Hence, the formation reaction of the ZVO is “self-limiting” in that the production of ZVO limits its further production by lowering the pH. Thus, considering solely the VOX cathode side, the formation of ZVO is not sustainable.

However, the presence of the Zn metal counter electrode perturbs the aforementioned behavior of the VOX cathode and indeed renders the situation less favorable. The metallic Zn on the anode undergoes the following corrosion reaction and increases the pH of the electrolyte by taking up protons:

3 $${{{{{{\rm{Zn}}}}}}}_{({s})}+2\,{{{{{{\rm{H}}}}}}}_{(aq)}^{+}\to {{{{{{\rm{Zn}}}}}}}_{(aq)}^{2+}+{{{{{{\rm{H}}}}}}}_{2(g)}\uparrow$$

The increased pH of the electrolyte allows the system to shift back to the region in which VO_2_(OH)_2_^−^ exists and enables ZVO to form. Therefore, the formation of ZVO is no longer self-limiting. As schematically illustrated in Fig. 4c, the overall process in the full-cell can be understood such that the pH regulation associated with the balanced production and uptake of protons from both sides of the cell renders the formation of ZVO unstoppable, which must impair the cyclability of the cell.

The vanadium dissolution behavior across the pH range with respect to time is depicted in Fig. 4b. In the absence of the zinc metal counter electrode, the formation of ZVO lowers the pH of the electrolyte, but, the time that elapses during the pH change depends on the amount of electrolyte. More specifically, under lean electrolyte conditions a short amount of time is required (trajectory i), whereas in the case of excess electrolyte it takes much longer (trajectory ii). This difference in the amount of time required for the pH change could affect the formation of ZVO. Along trajectory i, the concentration of the dissolved VO_2_(OH)_2_^−^ may not reach the critical value for the nucleation of the ZVO. By contrast, trajectory ii may provide sufficient time and the concentration of VO_2_(OH)_2_^−^ may be adequate to nucleate the ZVO phase. Contrary to these two cases, the presence of the zinc metal counter electrode allows the pH of the electrolyte to remain sufficiently high to enable the ZVO phase to form persistently (trajectory iii), regardless of the amount of electrolyte. Thus, the zinc metal anode neutralizes the “self-limiting” behavior of the ZVO formation.

A simple experiment was carried out to validate the above interpretation (Supplementary Fig. 9a). Specifically, we monitored the pH of three different samples prepared in vials containing a fixed amount of the electrolyte; these samples consisted of a small amount of V_2_O_5_ (sample X), a large amount of V_2_O_5_ (sample Y), and a large amount of V_2_O_5_ together with metallic Zn powder (sample Z). With time, the pH of sample X decreased from 3.94 to below 3.0 after 96 h (Supplementary Fig. 9b). In comparison, the pH of sample Y changed much faster, taking only 12 h for the same pH reduction (Supplementary Fig. 9c). Unlike these two cases, the pH of sample Z did not change even after 10 days (Supplementary Fig. 9d). This control experiment fairly simulates the pH change behavior of actual full-cells under different conditions and supports our interpretation. In addition, sample Z′ in which only zinc metal was immersed in the same electrolyte was prepared and compared with sample Z (Supplementary Fig. 10). The hydrogen evolution was far more vigorous in sample Z than in sample Z′, which reconfirms that the co-presence of Zn metal and V_2_O_5_ accelerates the acidic corrosion, which is accompanied with hydrogen evolution.

### Generalization of fading mechanism

Having noted that the formation of ZVO is the common origin of capacity fading of the majority of vanadium-based cathodes in AZIBs, a cathode consisting of V_6_O_13_ (V613), a well-known^16^ cathode material for AZIBs, was tested in the same context. The XRD pattern (Supplementary Fig. 11a) validates that the V613 existed as a monoclinic crystalline phase (PDF-01-072-3893). The differential capacity curves of the VOX and V613 differ in terms of the number of peaks and their positions (Supplementary Fig. 11b, c), suggesting different Zn storage mechanisms for the two materials. However, similar to VOX, V613 also experienced severe capacity loss of 34% after 50 cycles at the current density of 200 mA g^−1^ (Supplementary Fig. 11d). Supplementary Fig. 12 displays the ex-situ XRD patterns of the discharged V613 electrode in the first and 50^th^ cycles. After the first discharge, only the peaks corresponding to the by-product BZS were observed for the V613 electrode, whereas after the 50^th^ discharge, BZS peaks with lower intensity and ZVO peaks with higher intensity were simultaneously observed for the discharged electrode. The surface of the V613 electrode after the 50^th^ charge was covered with by-products with a sheet-like morphology, unlike the pristine electrode (Supplementary Fig. 13a, b). Given that the BZS disappears during the charge process (Supplementary Fig. 2a), this by-product must be ZVO. Moreover, consistent with the VOX case, the dissolution test of the charged V613 electrode performed in the lean electrolyte system (coin-cell) for five days indicated that the formation of the ZVO phase is feasible only in the presence of the zinc metal counter electrode (W/Zn) (Supplementary Fig. 14a). In addition, according to the GCD test, the ZVO-covered V613 (W/Zn) electrode retained only 69% of the capacity of the electrode that was not subjected to the dissolution test (no rest) (Supplementary Fig. 14b). Therefore, as with VOX, the dominant degradation mechanism of V613 is the formation of the ZVO on the surface of the electrode. Once again, the presence of the Zn metal anode inhibits the “self-limiting” behavior of the ZVO by regulating the pH, and could thus be the universal origin of the capacity degradation of vanadium-based cathodes from which vanadium dissolution is viable.

### Pairing with non-corrosive Zn metal anode

**Fig. 5:**
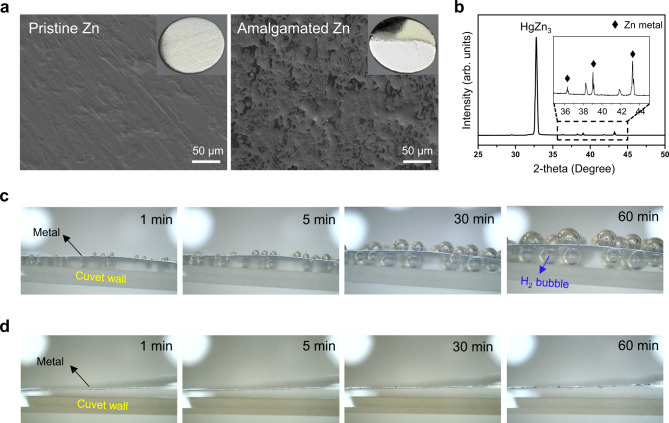
Preparation of amalgamated Zn and its corrosion resistance. **a** SEM images of pristine Zn metal and amalgamated Zn metal. The insets are optical photographs of the corresponding metals. **b** XRD pattern of as-prepared amalgamated Zn metal. **c**, **d** Magnified digital photographs of metal electrodes in 0.01 M HCl_(*aq*)_ (=pH 2) after different periods of time. **c** Pristine Zn and **d** amalgamated Zn.

The observations thus far deliver the message that if the corrosion of the zinc metal anode could be prevented, the cycle life of an AZIB cell should improve markedly. To prove this rationale, the Zn metal anode was replaced by its amalgamated Zn counterpart, a Zn-mercury alloy, by benchmarking the strategy in primary batteries. Although the use of mercury is not desirable due to its toxicity, amalgamated Zn was chosen because its resistance to corrosion is guaranteed^50^. This amalgamated Zn was prepared by a chemical conversion reaction^51^, details of which are provided in the methods. The amalgamated Zn had a mirror-like surface with a microscopic morphology that differed from that of the pristine zinc metal (Fig. 5a). Figure 5b presents the XRD pattern of the as-prepared amalgamated zinc. A new intense peak that was not present in the pristine zinc metal (Supplementary Fig. 15a) was discovered at 2θ ~32.7°, and this peak is well indexed to the HgZn_3_ alloy phase^52^. SEM-EDS mapping (depicted in Supplementary Fig. 15b) also shows that both mercury and zinc are evenly distributed throughout the surface of the amalgamated zinc electrode. Hence, these results confirmed that the amalgamated zinc metal was prepared properly with uniform phase distributions. The corrosion resistance of the pristine zinc metal and amalgamated zinc metal was directly compared by immersing both metals in hydrochloric acid and monitoring the evolution of hydrogen gas over time. In the case of the pristine zinc metal, hydrogen gas bubbles increasingly evolved with time (Fig. 5c, Supplementary Fig. 16a), whereas hydrogen gas evolution did not occur on the amalgamated zinc metal at all (Fig. 5d, Supplementary Fig. 16b).

**Fig. 6:**
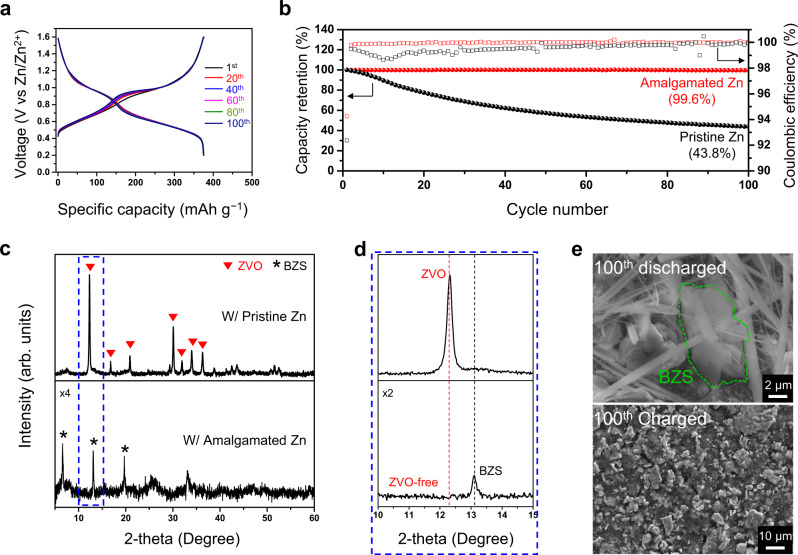
The influence of the non-corrosive Zn anode on the degradation of VOX electrode. **a** Galvanostatic charge-discharge profiles of VOX electrode at different cycle numbers when cycled at 200 mA g^−1^ with amalgamated zinc metal. **b** Cycling performance of VOX electrodes with pristine zinc metal and amalgamated zinc metal at 200 mA g^−1^. **c** Ex-situ XRD patterns of discharged VOX electrodes after 100 cycles when paired with pristine Zn metal and amalgamated Zn metal. **d** Magnified view of (**c**) in the 2θ range from 10°~15°. **e** SEM images of VOX electrodes at the 100^th^ cycle when paired with amalgamated zinc metal anode.

GCD tests with a full-cell in which a VOX cathode was paired with an amalgamated zinc metal anode were performed at a current density of 200 mA g^−1^. The specific capacity and the differential capacity curve of the amalgam-based full-cell were almost identical to those of the full-cell based on pristine zinc (Fig. 6a, Supplementary Fig. 17). However, the capacity retention of the amalgam-based full-cell after the first 100 cycles was 99.6%, which is far higher than the 43.8% of its pristine Zn-based counterpart (Fig. 6b). To verify that the improvement in the cyclability originates from the suppressed formation of the ZVO phase, the discharged VOX cathodes were analyzed using XRD after 100 cycles (Fig. 6c, d). For the VOX electrode in the amalgam-based full-cell, only peaks assigned to BZS were observed; the peaks corresponding to the ZVO were not observed at all. This is in contrast to the VOX paired with the pristine Zn anode, which showed intense peaks corresponding to the ZVO phase. The SEM images of the VOX electrodes after 100 cycles (Fig. 6e) support these results and show that no by-products exist on the surface of the charged electrode and that only BZS is present on the surface of the discharged electrode. In addition, the ex-situ XRD patterns of the amalgamated zinc after 100 cycles indicate that the peak corresponding to the alloy phase retained its high intensity (Supplementary Fig. 18). The more sustainable nature of the amalgamated zinc metal was also revealed by long-term symmetric cell tests (Supplementary Fig. 19). The symmetric cell consisting of the amalgamated zinc metal showed much longer cycle life with far smaller overpotentials. These results represent long-term corrosion resistance, and consequently, the formation of the ZVO is effectively suppressed toward markedly improved cyclability. The improvement of the cycling performance of the VOX cathode paired with the amalgamated zinc metal was also valid when tested at a high current density (Supplementary Fig. 20); when cycled at 5 A g^−1^, the cell preserved 94.4% of its original capacity after 7000 cycles whereas the cell based on the pristine zinc metal retained only 24.3% after the same number of cycles with the same operating conditions. Importantly, we learned that the capacity fading depends highly on the choice of Zn salt. Particularly, the use of 3 m ZnSO_4(*aq*)_, instead of 3 m Zn(OTf)_2(*aq*)_, as the electrolyte did not enhance the cyclability as much even for the amalgam-based full-cell (Supplementary Fig. 21). This is because the degradation mechanism in the aqueous electrolyte based on zinc sulfate is not relevant to the formation of the ZVO phase (Supplementary Fig. 22b, d). Collectively, these results provide the lesson that the degradation of a cell needs to be viewed comprehensively by considering the reactions on the electrodes on each side of the cell as well as the cross-communication between them. Notably, the electrochemical performance of ZnSO_4(*aq*)_ is known to be inferior to that of Zn(OTf)_2(*aq*)_ even though ZnSO_4(*aq*)_ hardly induces the formation of the ZVO phase (Supplementary Fig. 22a, c). This tendency is attributed to the higher Zn^2+^ desolvation energy of ZnSO_4(*aq*)_ that results in higher charge-transfer resistance as well as the relatively higher affinity of the SO_4_ anion for water molecules, which induces the dissolution of vanadium oxides^44,46^. Additionally, it is anticipated that the BZSs produced in different electrolytes, Zn_12_(CF_3_SO_3_)_9_(OH)_15_·nH_2_O in Zn(OTf)_2(*aq*)_ and Zn_4_(SO_4_)(OH)_6_·5H_2_O in ZnSO_4(*aq*)_, affect the cyclability of the corresponding cells^47,53^.

In summary, we pinpointed the electrochemically inactive zinc pyrovanadate (ZVO) phase, formed by dissolution-precipitation reactions, as the main origin of the capacity fading of VOX electrodes. Moreover, the formation of ZVO on the VOX electrode is not simply an event restricted to the cathode; rather, it is related to the corrosion at the Zn metal anode, which perturbs the pH of the electrolyte and therefore stops the “self-limiting” nature of the VO_2_(OH)_2_^−^ dissolution. A series of experiments with different amounts of electrolyte in the presence and absence of a Zn metal counter electrode verified our interpretation based on cross-communication between the anode and cathode, especially by taking into consideration the sensitivity to the pH of the electrolyte in relation to the corrosion at the Zn metal anode. The conspicuously improved cyclability by incorporating the corrosion-resistant amalgamated Zn anode reconfirmed the importance of the effect of the corrosion of the anode on the cycle life and offers a viable solution although the toxicity of mercury would have to be addressed. The findings of this study provide a plausible explanation for the unsatisfactory performance reported for many AZIBs based on vanadium oxides during their cycling at low current densities and following long-term storage. Furthermore, consideration of the cross-communication between both electrodes as an important parameter to determine the key factors that affect the performance of a cell is expected to serve as a stepping-stone towards advancing AZIBs to becoming a reliable option for large-scale energy storage systems.

## Methods

### Materials preparation

V_2_O_5_ (Aldrich, 99.6%), 30 wt% H_2_O_2(*aq*)_ solution (Aldrich), Zn(OTf)_2_ (Aldrich, 98%), ZnSO_4_·7H_2_O (Aldrich, 99%), HgCl_2_ (Aldrich, 99.5%), 1N HCl_(*aq*)_ solution (SAMCHUN) and V_6_O_13_ (Alfa Aesar, 99.5%) were purchased and used without further purification. VOX was synthesized based on a sol-gel process without any thermal treatment.^54^ 0.25 g of V_2_O_5_ was first added to a mixture of deionized water (3.85 g) and hydrogen peroxide solution (30 wt%, 1.15 g). After stirring for 15 minutes, 20 g of deionized water was added, and the solution was thoroughly mixed again. The homogeneous dark red solution was then sonicated for 2 h, followed by freeze drying to complete the synthesis of VOX gel. The as-synthesized VOX and as-received V613 were ground to a fine powder before using them as cathode active materials. The amalgamated zinc was prepared by dipping the zinc metal foil into an amalgamation solution (aqueous solution of 0.1 M HgCl_2_ and 0.1 M HCl). After the amalgamation, the amalgamated zinc was washed with deionized water to remove residues.

### Characterization

XRD patterns of the powder and electrode films were obtained using a Bruker D8 Advance diffractometer equipped with a Cu Kα source (λ = 1.5418 Å). The diffraction data were collected in the 2θ range from 5° to 60° with a step size of 0.02° and an acquisition time of 0.5 seconds per point. XRD analysis was conducted at the Research Institute of Advanced Materials (RIAM) at Seoul National University. SEM images were obtained using a FE-SEM 7800F Prime microscope (JEOL Ltd., Japan) equipped with an EDS attachment. XPS measurements were performed using an AXIS SUPRA spectrometer (Kratos, UK). SEM and XPS analyses were performed at the National Center for Inter-university Research Facilities (NCIRF) at Seoul National University. All electrode samples for ex-situ analyses were disassembled and washed with deionized water several times after cycling to remove the residues. TGA analysis was conducted to measure the water content in the VOX using a Discovery TGA (TA Instruments, USA) under N_2(*g*)_ atmosphere. The pH was measured using an Orion Star A215 pH meter (Thermo Scientific).

### Electrochemistry

To fabricate the cathodes, slurries were first prepared by mixing the active material, conductive carbon (Super P) and poly(vinylidene fluoride) (PVdF, Kynar) binder with a weight ratio of 7:2:1 in *N*-methyl-2-pyrrolidone (NMP, Aldrich, 99.5%). These slurries were then coated onto stainless steel (SUS) foil and dried at 70 °C for 12 h. The mass loading was 1.0–1.5 mg cm^−2^. Zinc foil (Goodfellow, 0.05 mm thick) and glass fiber membrane (GF/C, Whatman) were used as the anode and separator, respectively. Full cells were fabricated in CR2032-type coin cells (SUS316L, Wellcos, Korea) and ~0.1 g of 3 m Zn(OTf)_2(*aq*)_ electrolyte was injected into each coin cell. The assembled cells were tested using a WBCS3000L battery cycler (WonATech, Korea) at 25 °C. The specific capacities were calculated with respect to the mass of the active material. In the GCD tests, after 6 h of rest, the first discharge was conducted at the current density of 100 mA g^−1^ to activate the cell, and the following cycles were performed at 200 mA g^−1^ or 5 A g^−1^ in constant current (CC) mode for both charging and discharging. Full-cells were cycled in the voltage window of 0.2–1.6 V. The symmetric cell tests of the zinc metal were conducted at the current density of 1 mA cm^−2^ with the areal capacity of 1 mAh cm^−2^.

## Supplementary information

Supplementary information

## Data Availability

The data that support the plots within this paper and other findings of this study are available from the corresponding author upon reasonable request.
