# Supplementary material for: Corrosion as the origin of limited lifetime of vanadium oxide-based aqueous zinc ion batteries
Source: Nat Commun. 2022 May 2;13:2371. doi: 10.1038/s41467-022-29987-x (PMC9061739; doi:10.1038/s41467-022-29987-x)
Supplement: Supplementary file 1 — Supplementary information [file 41467_2022_29987_MOESM1_ESM.pdf]

Supplementary information:

Corrosion as the origin of limited lifetime of vanadium oxide-based aqueous zinc ion batteries

Yangmoon Kim[1], Youngbin Park[1], Minkwan Kim[1], Jimin Lee[1], Ki Jae Kim[2]* and Jang Wook Choi[1]*

[1]School of Chemical and Biological Engineering and Institute of Chemical Processes, Seoul National University, 1 Gwanak-ro, Gwanak-gu, Seoul 08826, Republic of Korea

[2]Department of Energy Engineering, Konkuk University, Neungdong-ro 120, Gwangjin-gu, Seoul 05029, Republic of Korea

*Correspondence: kijaekim@konkuk.ac.kr (K. J. Kim), jangwookchoi@snu.ac.kr (J. W. Choi).

Supplementary Figures

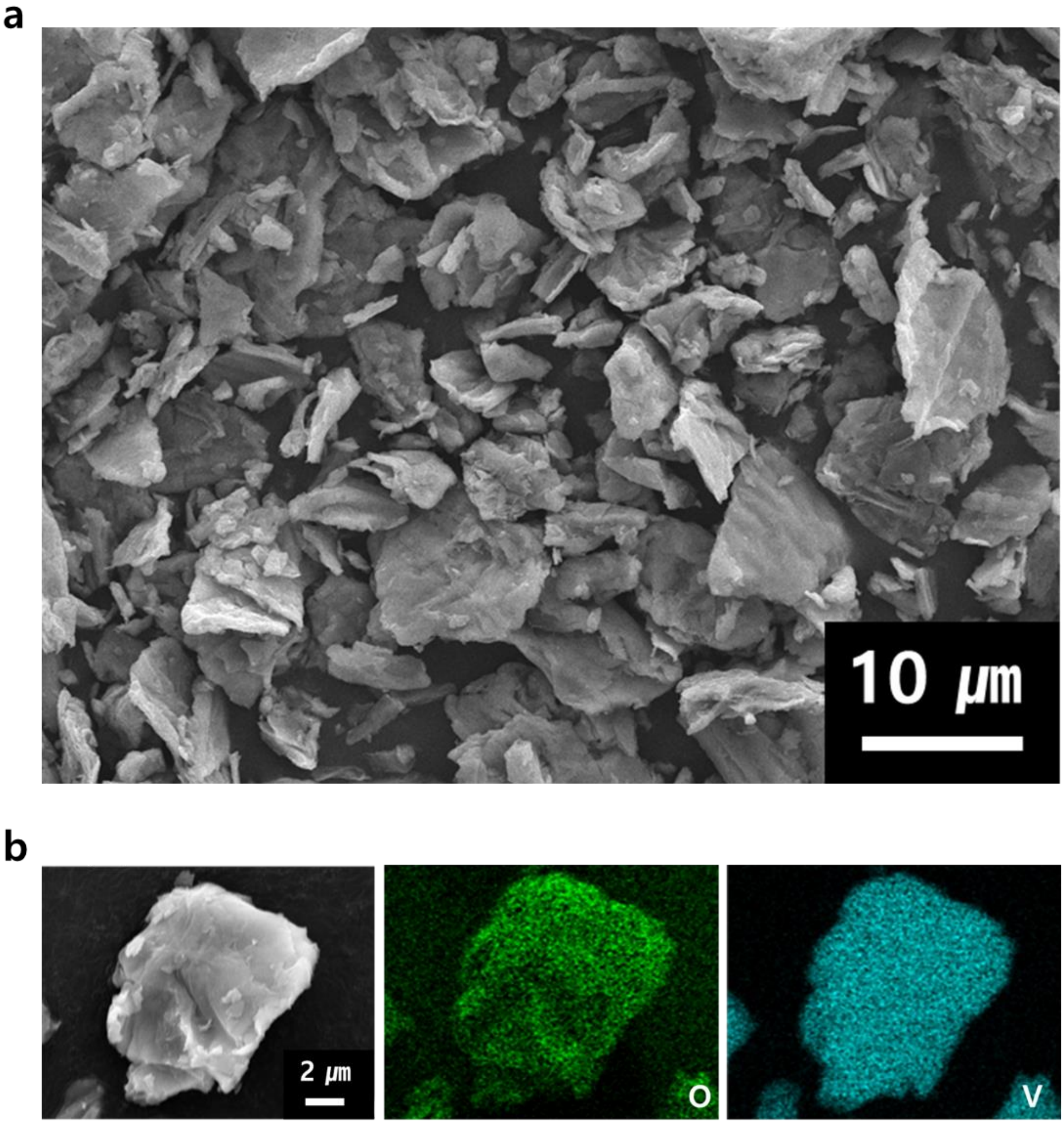

Supplementary Figure 1. SEM-EDS analysis of as-synthesized VOX. **a**, SEM image of as-prepared VOX. **b**, EDS mapping of as-prepared VOX.

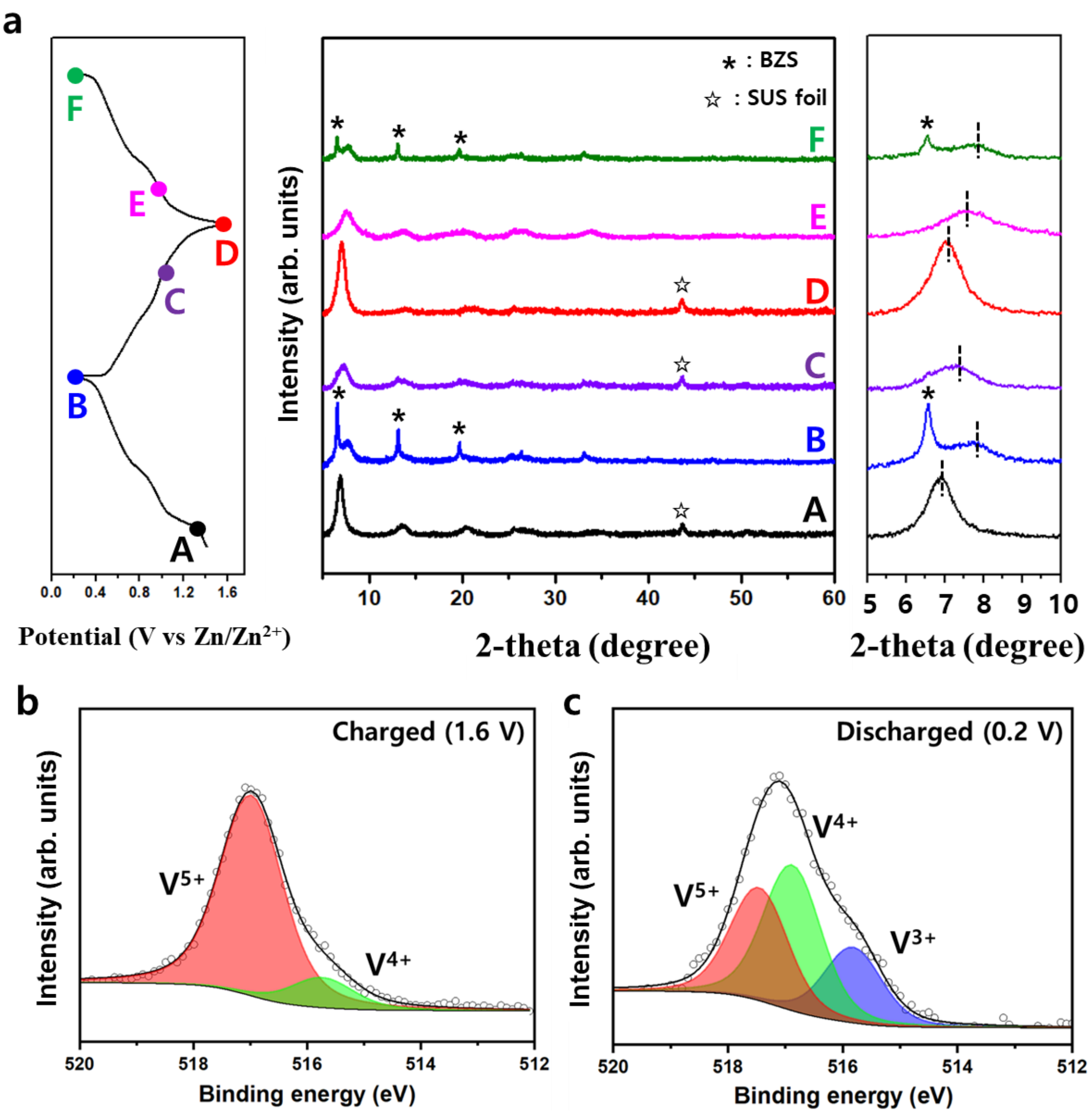

Supplementary Figure 2. Analysis of the electrochemical reaction mechanism of the VOX electrode. **a**, Ex-situ XRD patterns of the VOX electrode at selected states in the first cycle. XPS profiles of V $2p_{3/2}$ in the **(b)** fully charged state and **(c)** fully discharged state in the first cycle.

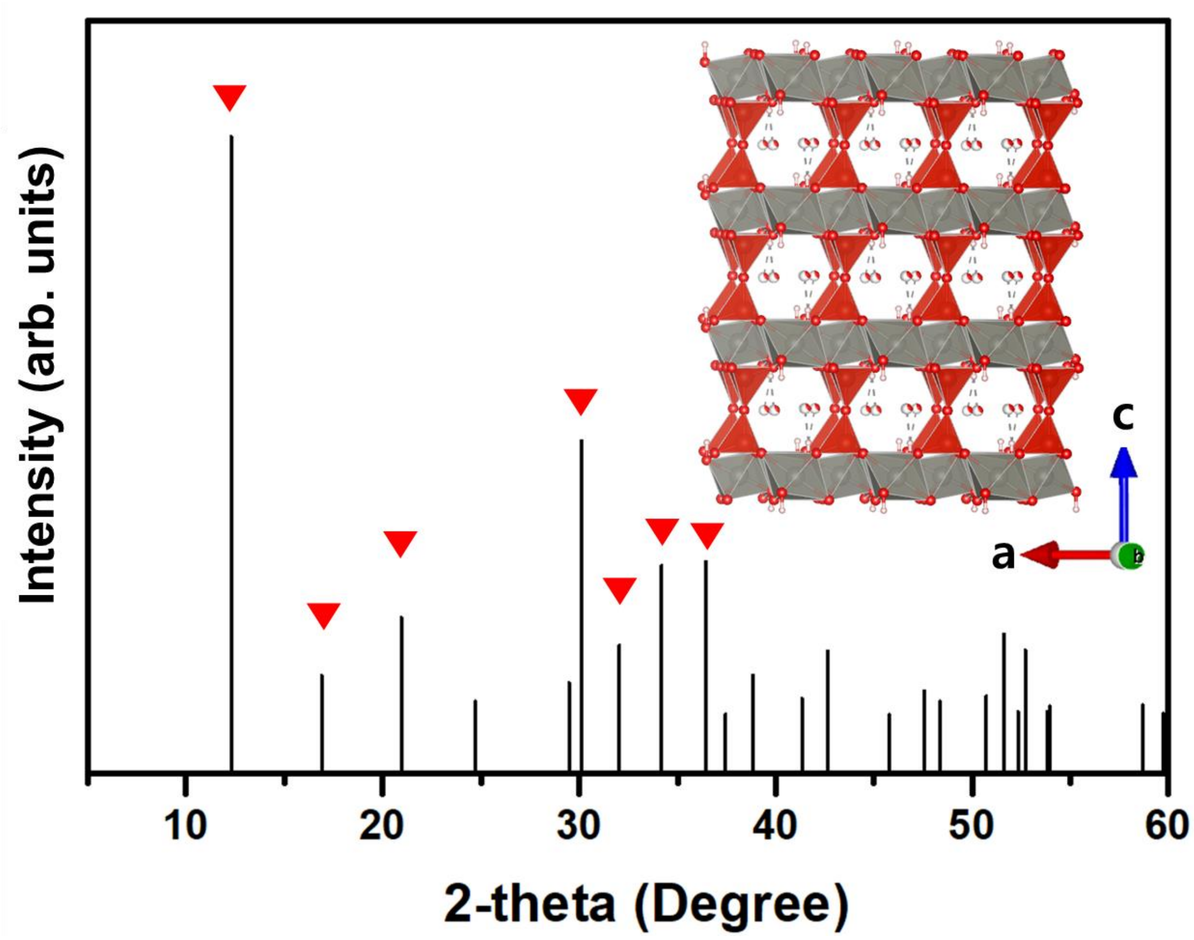

Supplementary Figure 3. XRD index of ZVO phase (PDF 01-087-0417). Inset: crystal structure of ZVO viewed along the b-axis.

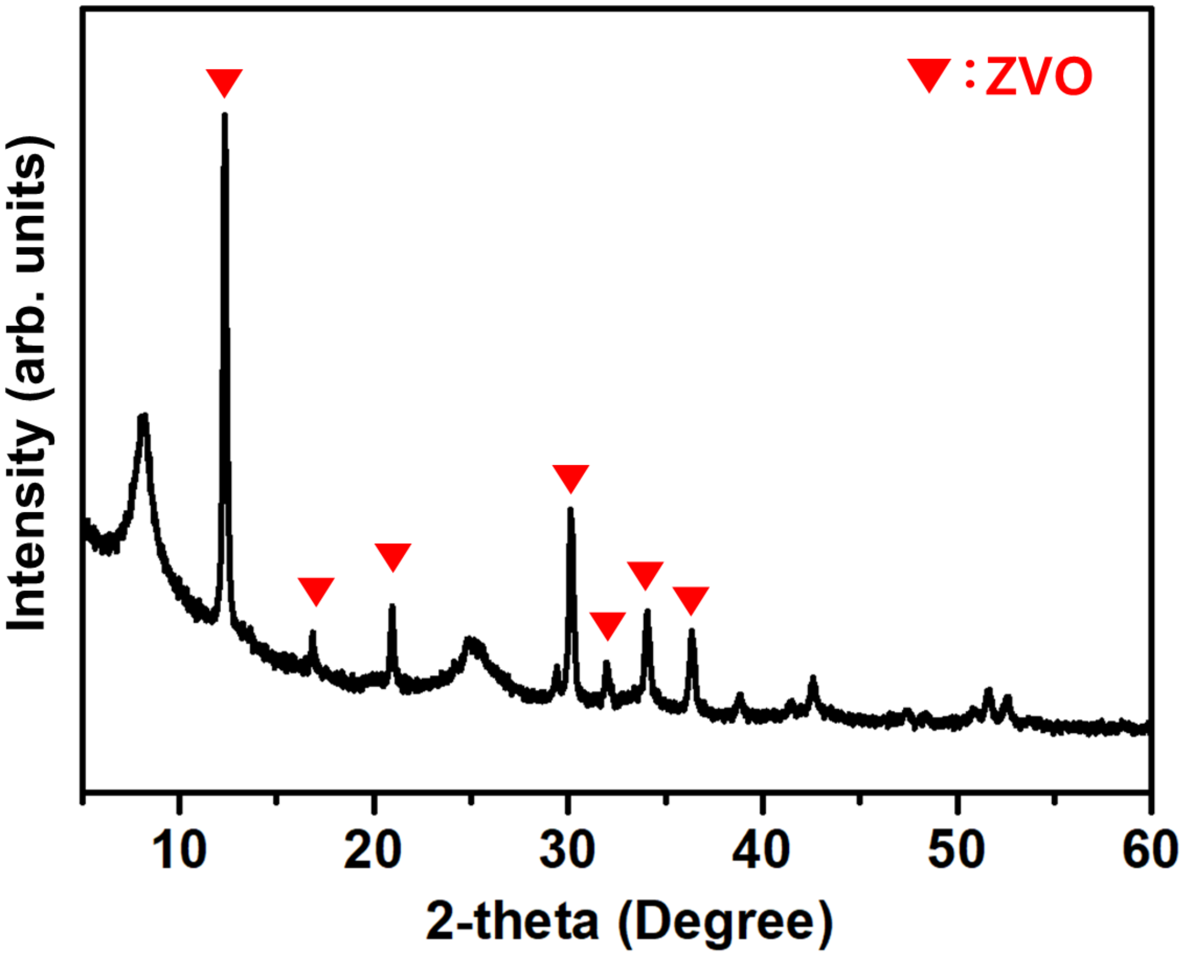

Supplementary Figure 4. Ex-situ XRD pattern of charged VOX electrode in the 50th cycle.

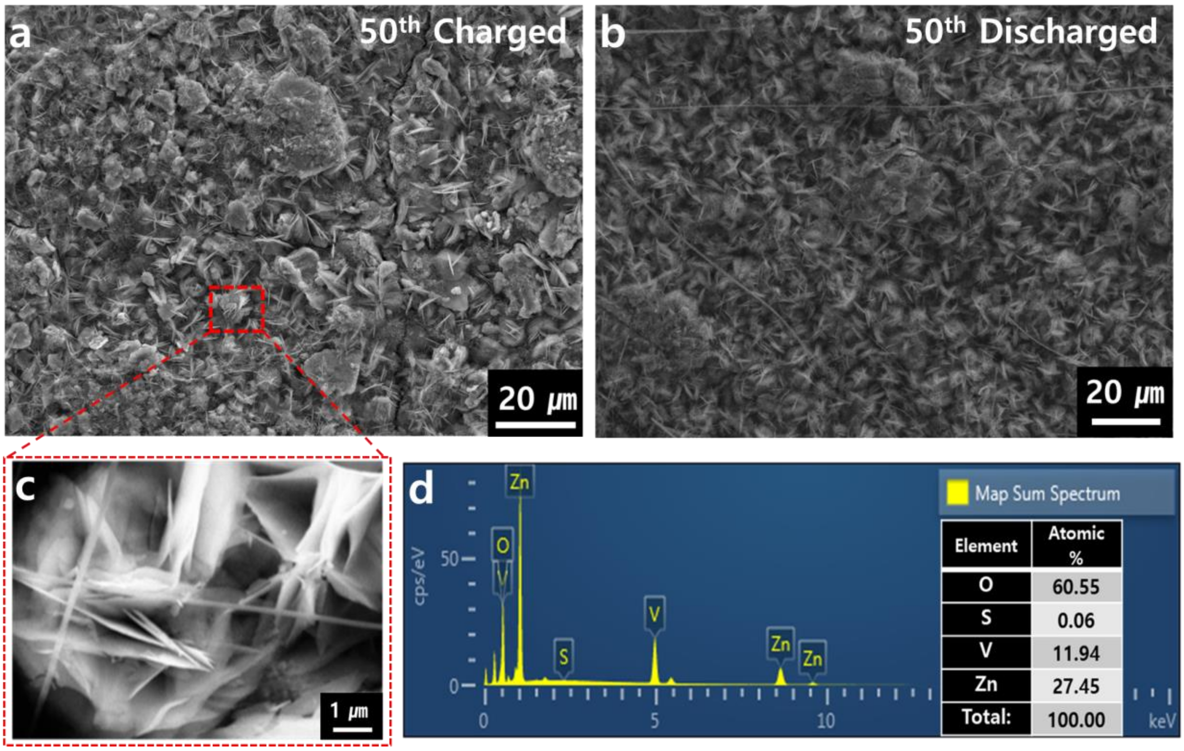

Supplementary Figure 5. SEM images of VOX electrode and ZVO phase in the 50th cycle. **a,** Fully charged state. **b,** Fully discharged state. **c**, ZVO phase on the surface of charged VOX electrode. **d**, EDS spectrum of **(c)**.

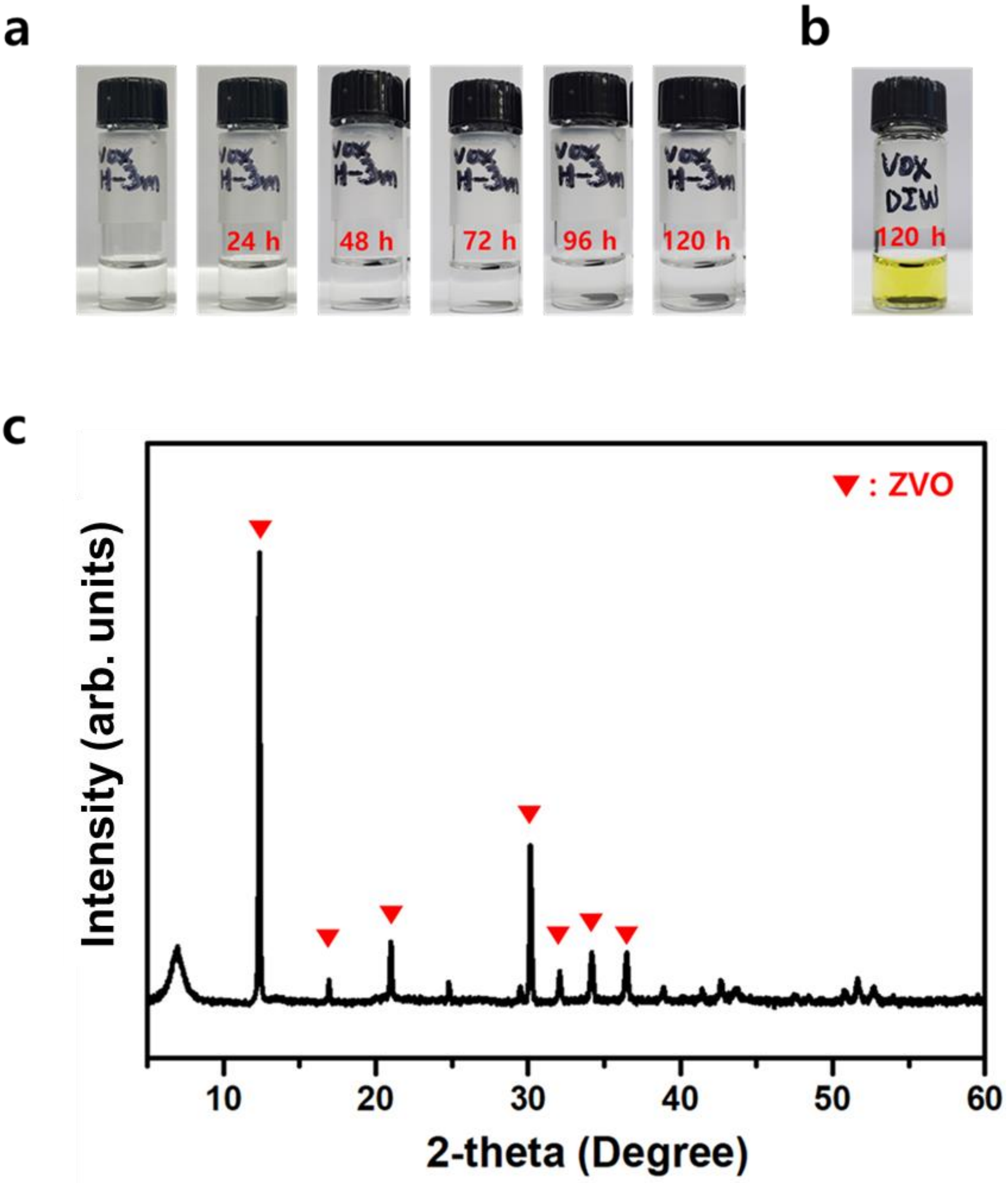

Supplementary Figure 6. Dissolution test of VOX electrode when immersed in an excess amount of electrolyte. a, Digital photographs of vials containing 3 m $Zn(OTf)_{2(aq)}$ and VOX electrodes after different periods of time. **b**, The same test as in **(a)** but in deionized water. **c**, XRD pattern of VOX electrode after 120 h of dissolution test in the electrolyte containing 3 m $Zn(OTf)_2$.

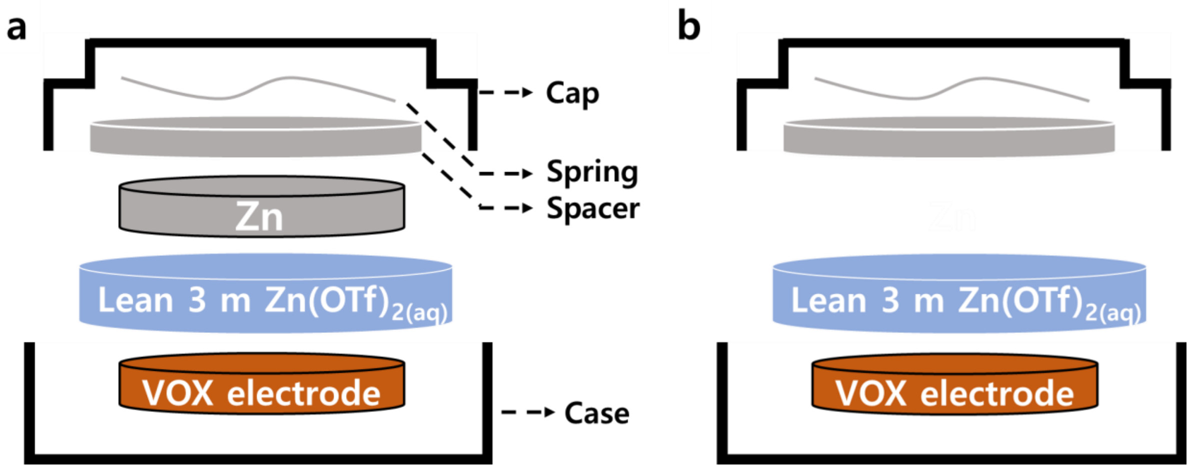

Supplementary Figure 7. Schematic illustration of coin cell configuration. a, The cell prepared with a zinc metal counter electrode (W/Zn). **b**, The cell without a zinc metal counter electrode (W/O Zn).

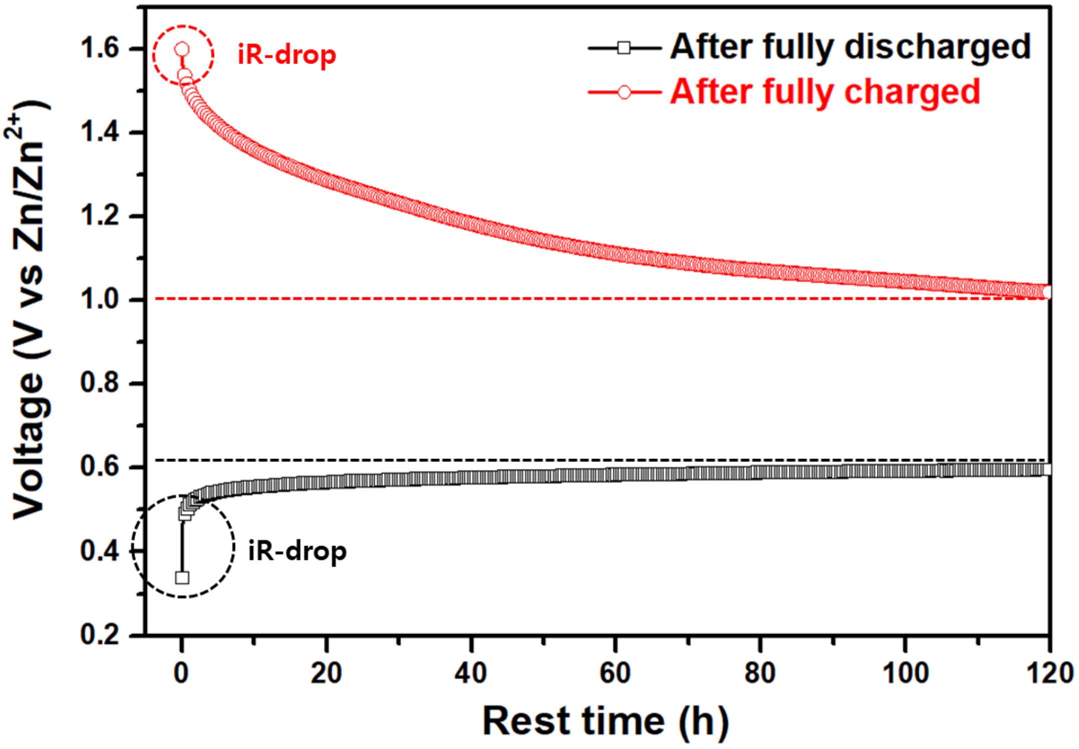

Supplementary Figure 8. OCV change of the fully charged and fully discharged VOX full-cells during five days of rest.

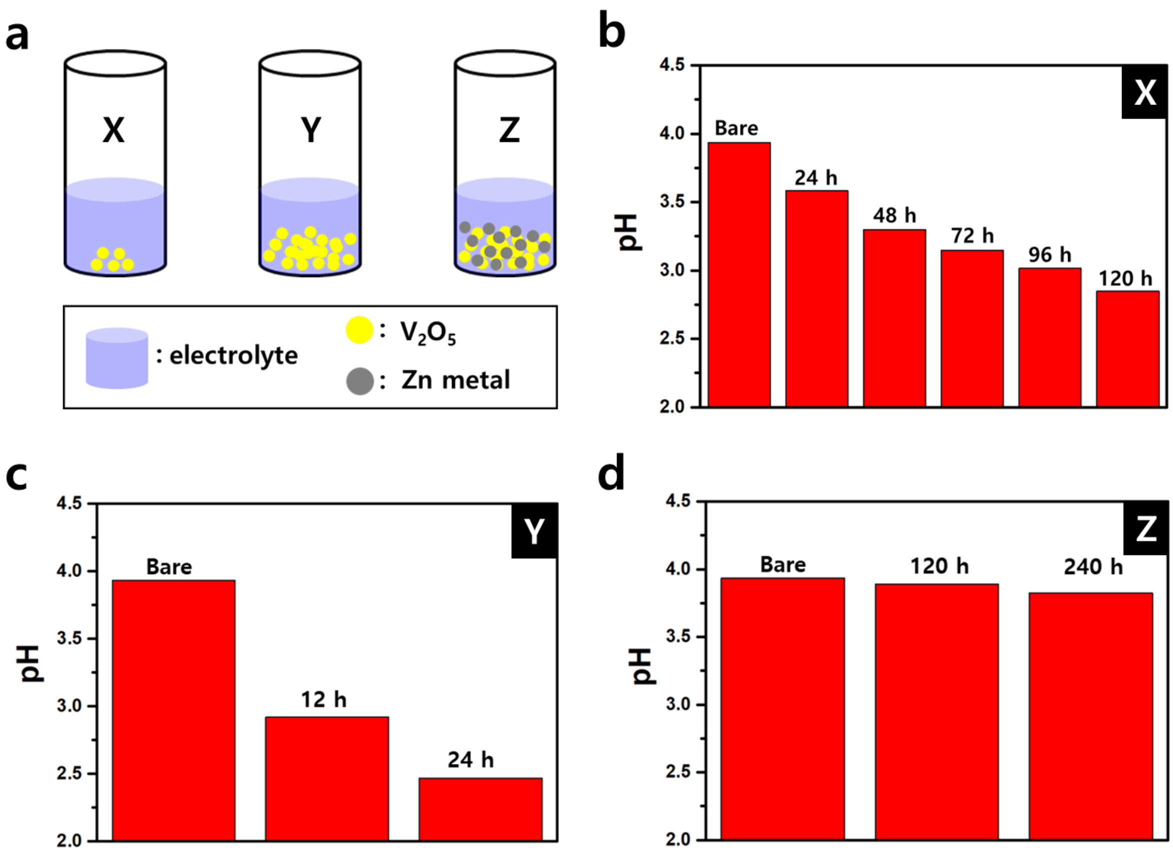

Supplementary Figure 9. Tests that simulate various dissolution conditions to examine the pH change. a, Schematic illustration of samples prepared to determine the rate at which the pH changes under individual dissolution conditions. X, Y, and Z represent the dissolution test conditions of "bulk electrolyte," "lean electrolyte," and "lean electrolyte with Zn metal," respectively. pH changes of **(b)** sample X, **(c)** sample Y, and **(d)** sample Z.

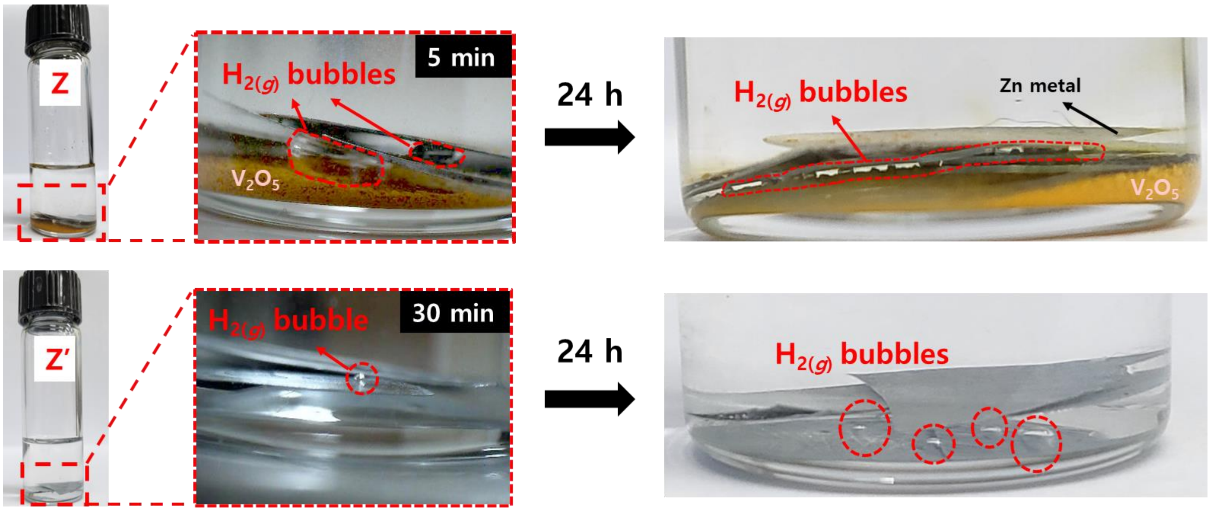

Supplementary Figure 10. The magnified digital photographs of hydrogen gas evolution in the sample Z and Z′.

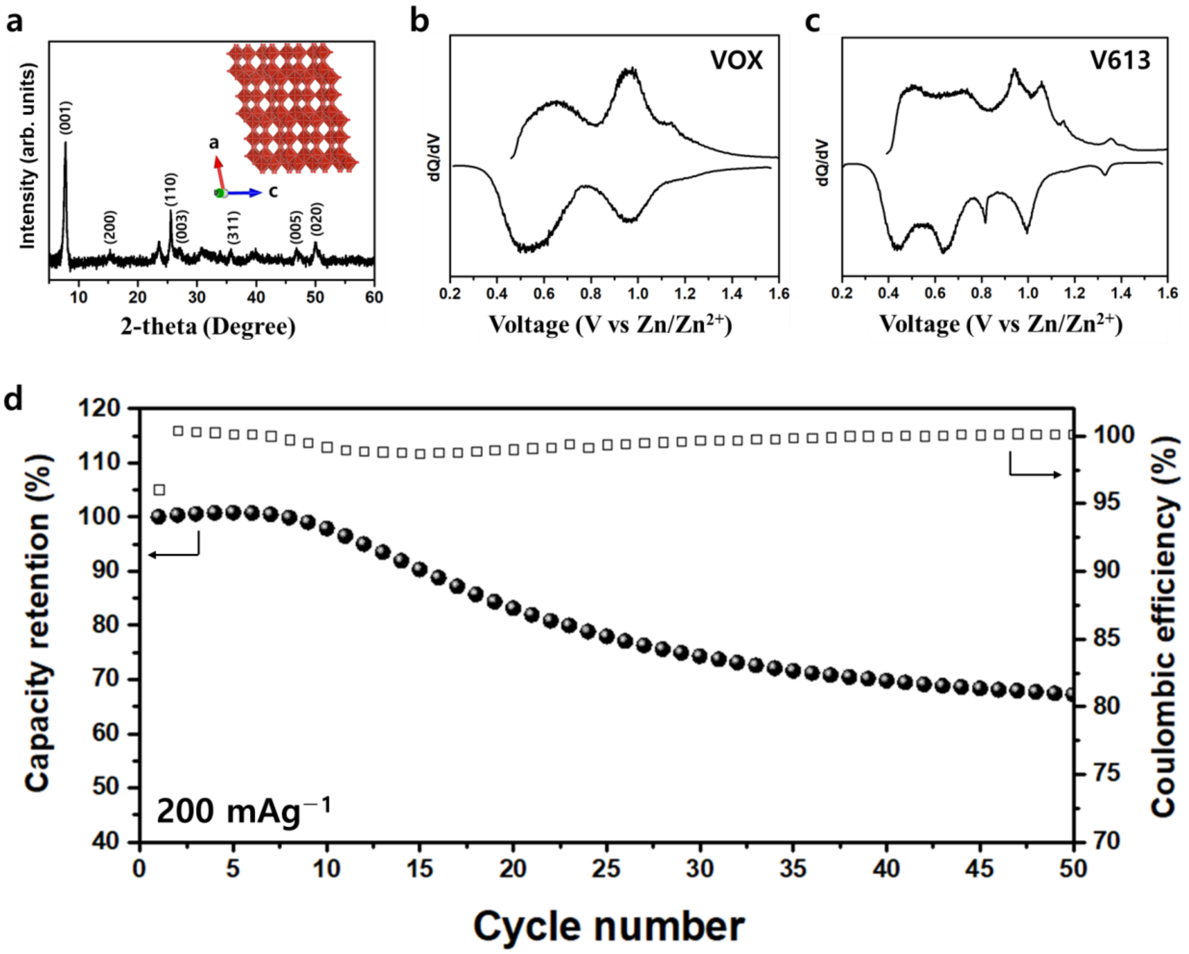

Supplementary Figure 11. Structure of V613 and its electrochemical behavior in 3 m $Zn(OTf)_{2(aq)}$. a, XRD pattern of V613. Inset: crystal structure of V613 viewed along the b-axis. Differential capacity plots of **(b)** VOX and **(c)** V613 in their first charge/discharge cycles at 200 mA g^{-1}. **d**, Cycling performance of V613 electrode when cycled at 200 mA g^{-1}.

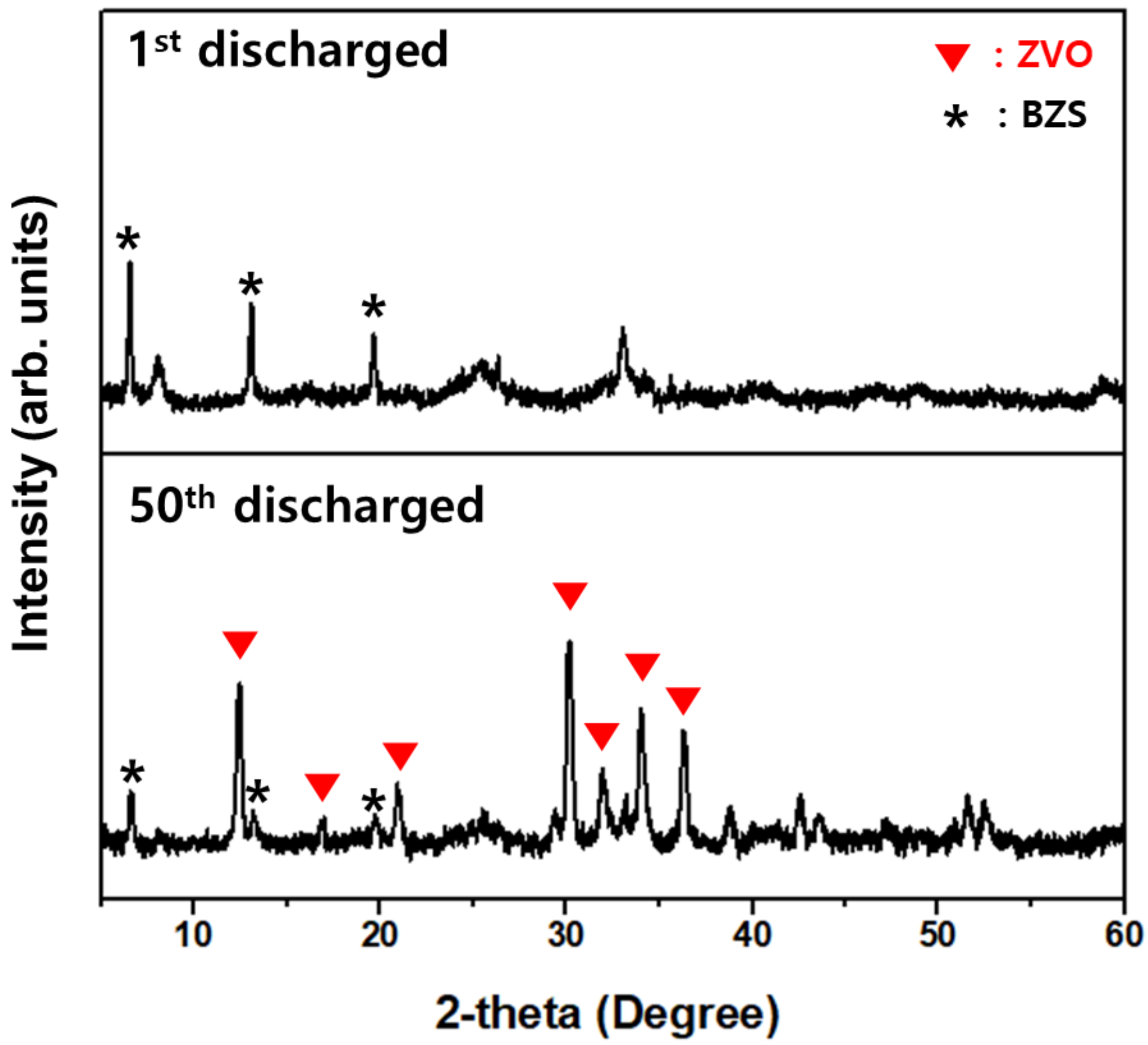

Supplementary Figure 12. Ex-situ XRD patterns of V613 electrodes at different cycling points.

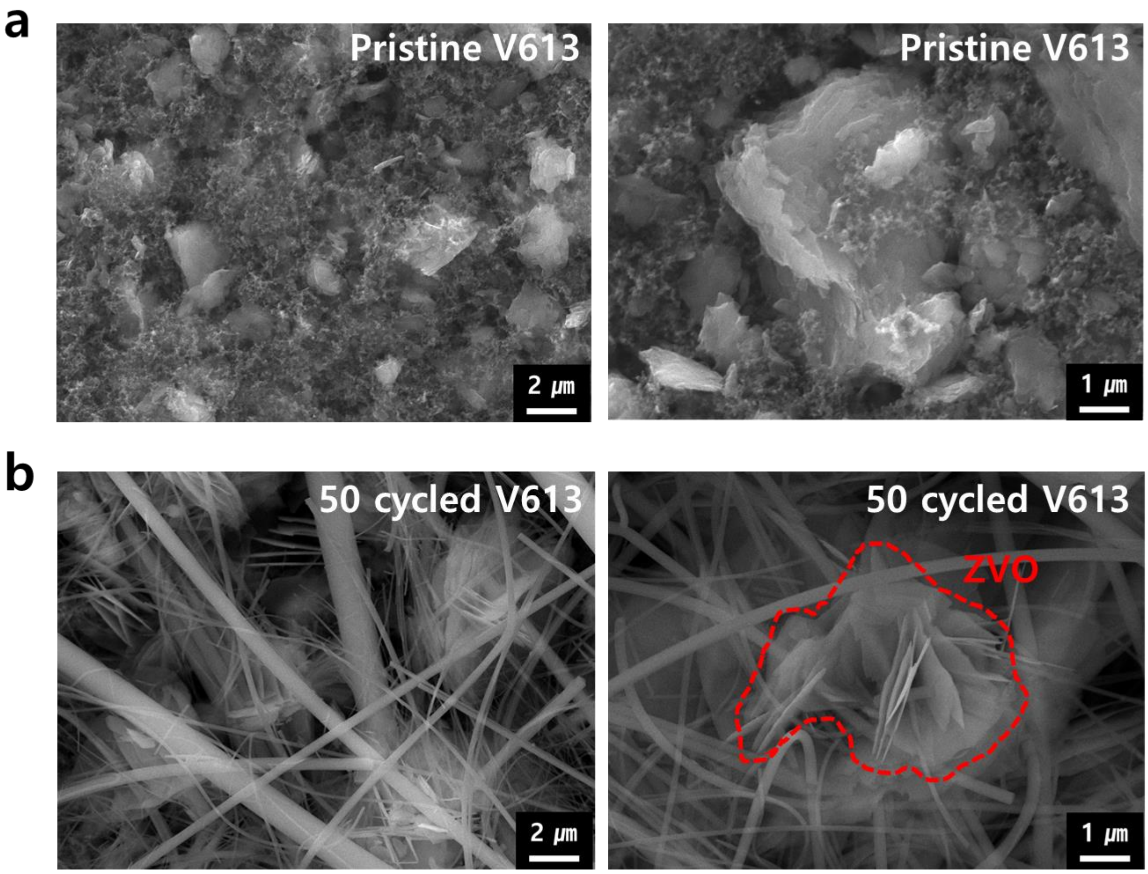

Supplementary Figure 13. SEM images of V613 electrodes. a, Pristine state. **b,** 50th charged state.

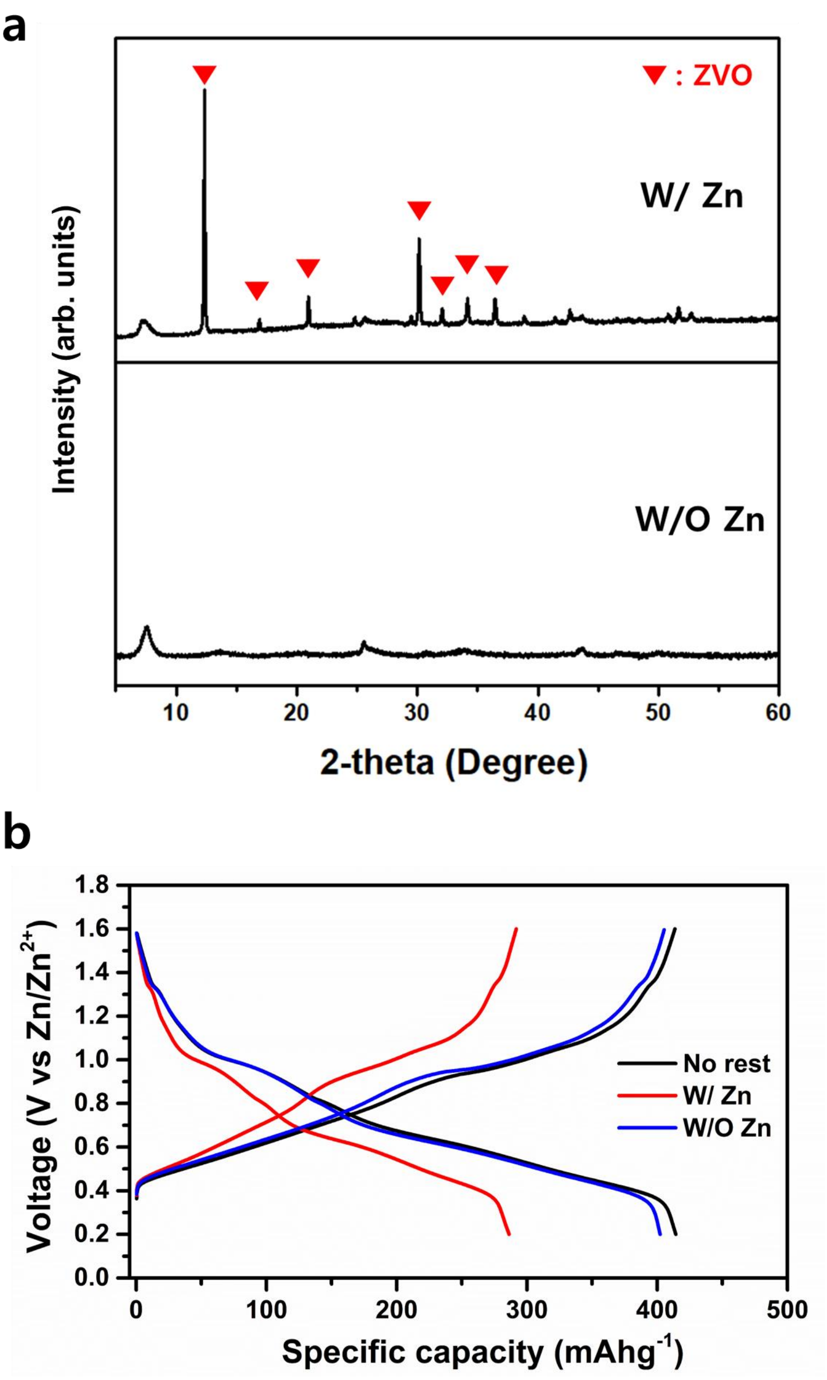

Supplementary Figure 14. Structural and electrochemical analyses of V613 under different conditions. a, Ex-situ XRD pattern of V613 electrodes after 5 days of rest in the charged state with and without the zinc metal counter electrode. **b**, First galvanostatic charge and discharge profiles of V613 electrodes under different test conditions.

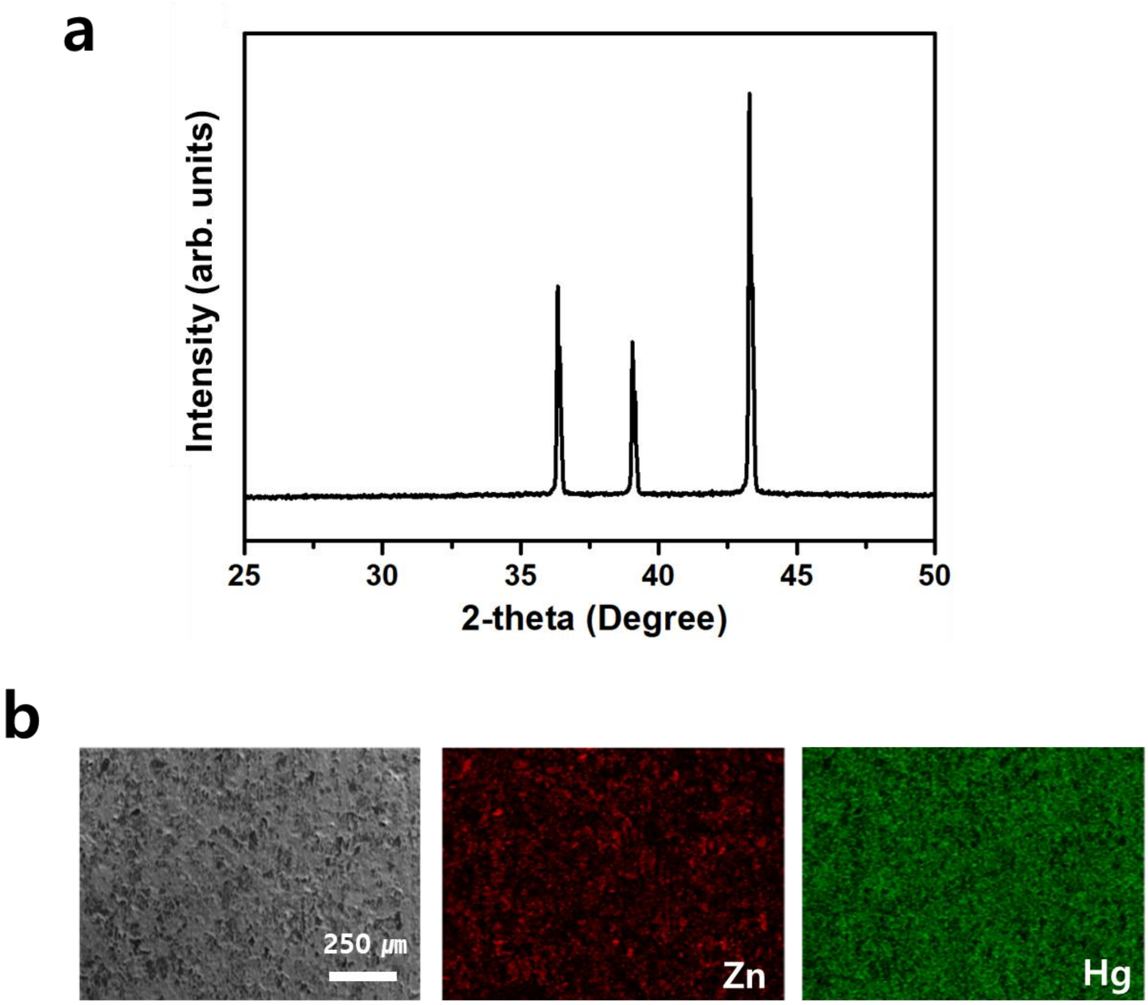

Supplementary Figure 15. Characterization of pristine and amalgamated zinc metal. a, XRD pattern of pristine zinc metal. **b**, SEM-EDS mapping of amalgamated zinc metal.

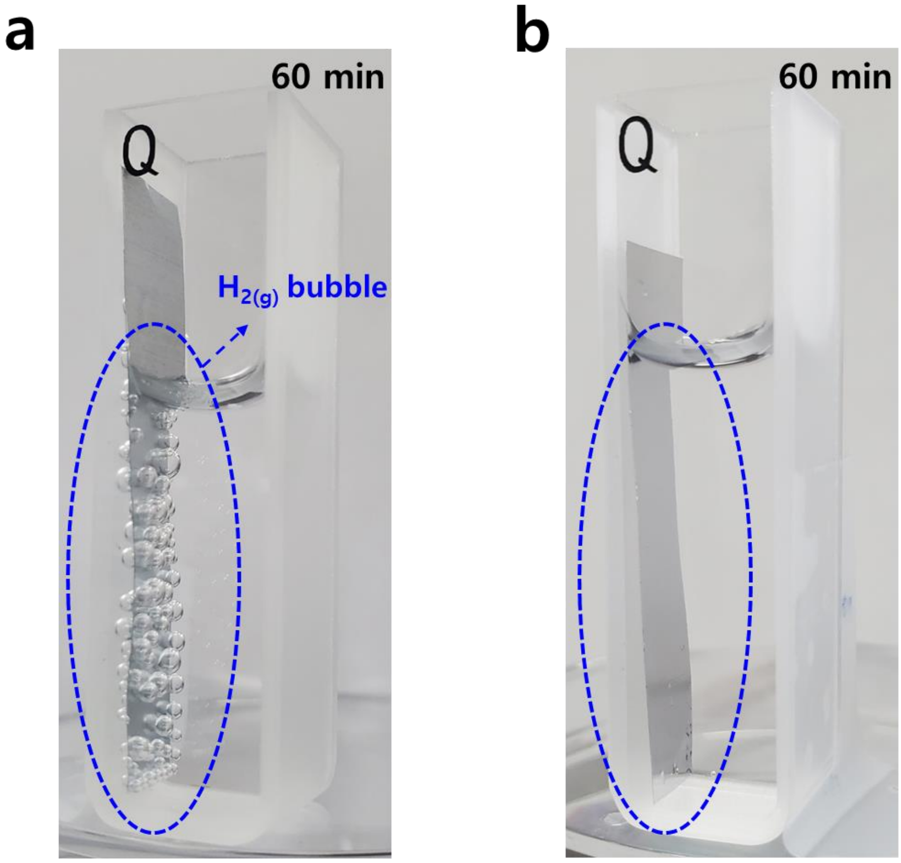

Supplementary Figure 16. Digital photographs of metal electrodes in 0.01 M $HCl_{(aq)}$ (= pH 2) after 60 min. a, Pristine zinc metal. **b,** Amalgamated zinc metal.

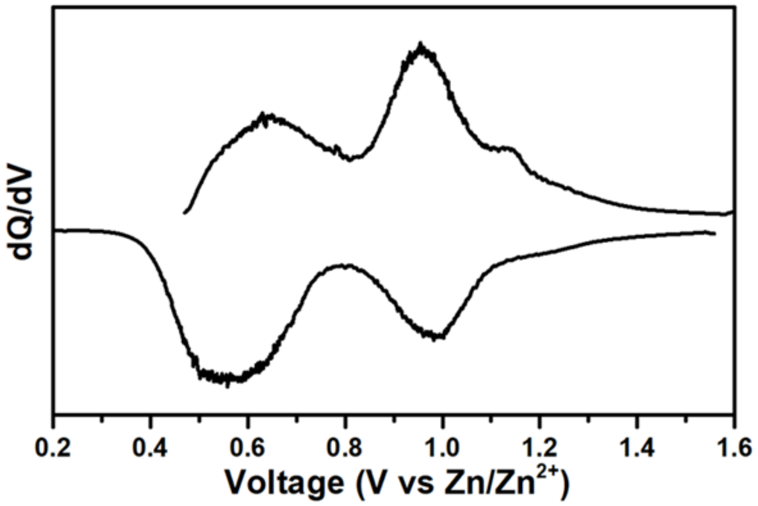

Supplementary Figure 17. Differential capacity curve of VOX when paired with amalgamated zinc metal anode.

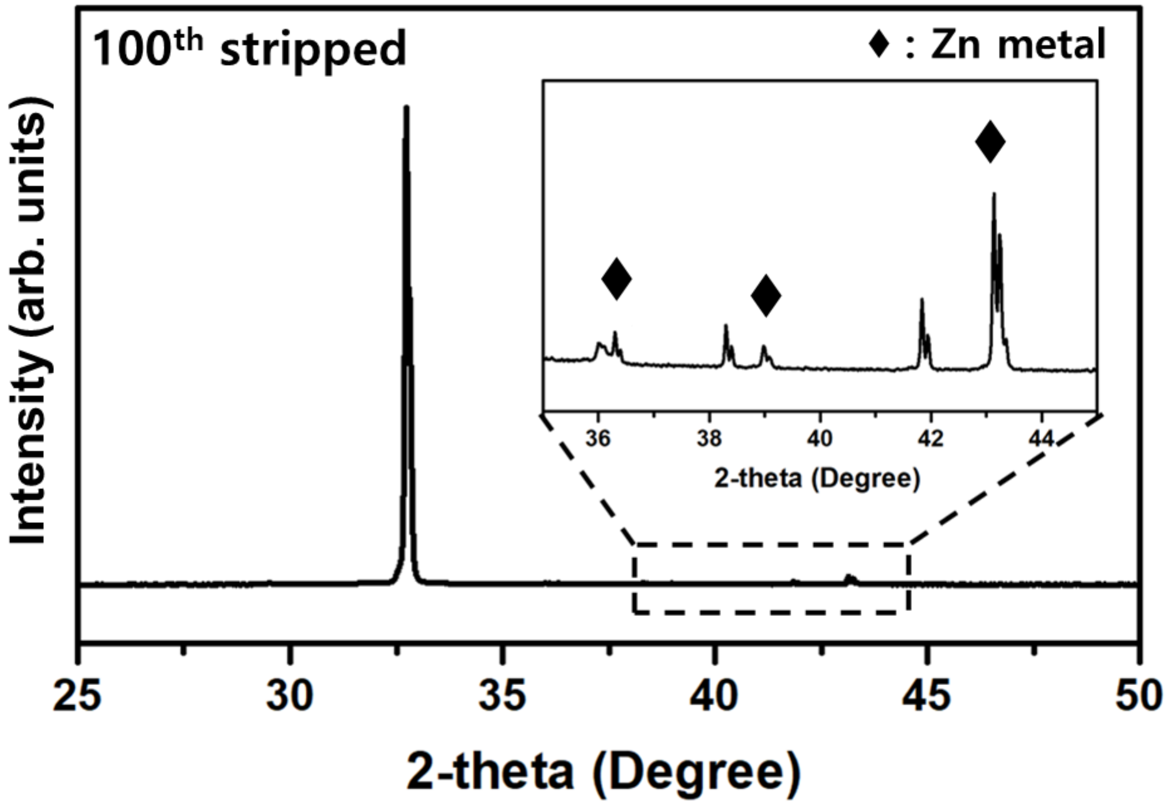

Supplementary Figure 18. Ex-situ XRD pattern of amalgamated zinc metal anode after Zn stripping in the 100th cycle when cycled at 200 mA g^{-1}.

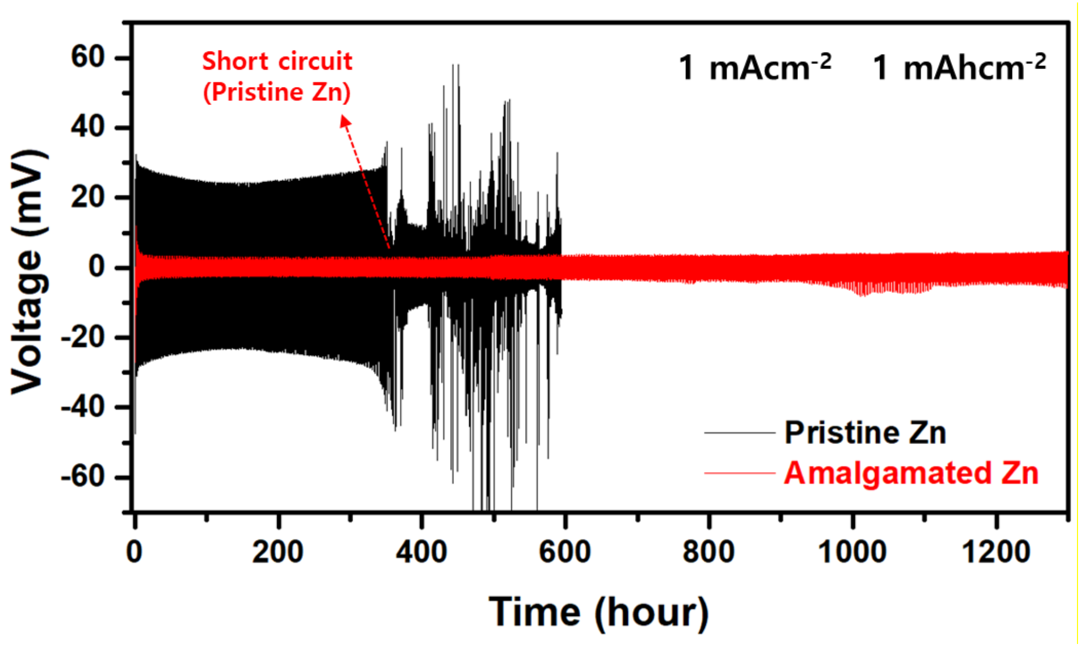

Supplementary Figure 19. **Long-term symmetric cell tests at the current density of 1 mA cm^{-1} and the areal capacity of 1 mAh cm^{-1}.**

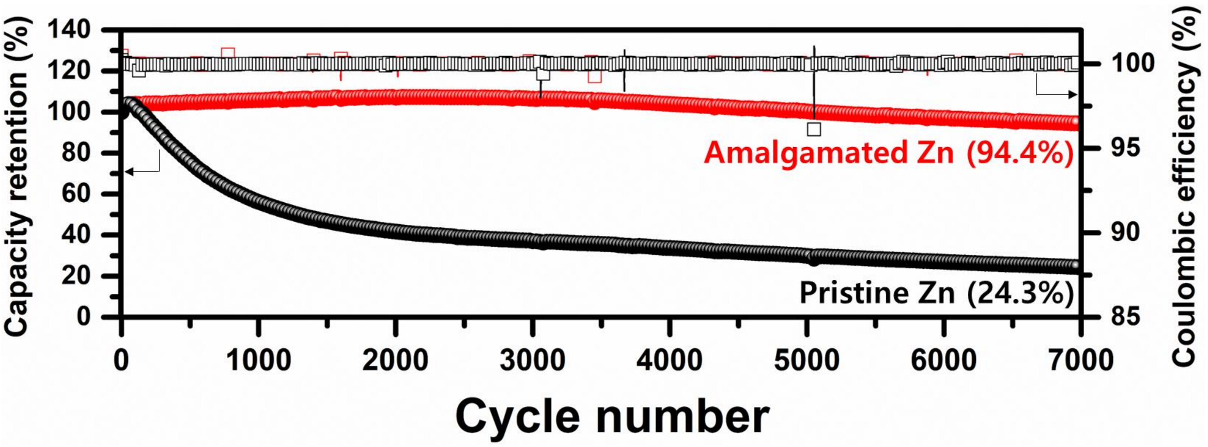

Supplementary Figure 20. **Cycling performance of VOX electrodes with the pristine zinc metal and amalgamated zinc metal when cycled at 5 A g^{-1}.**

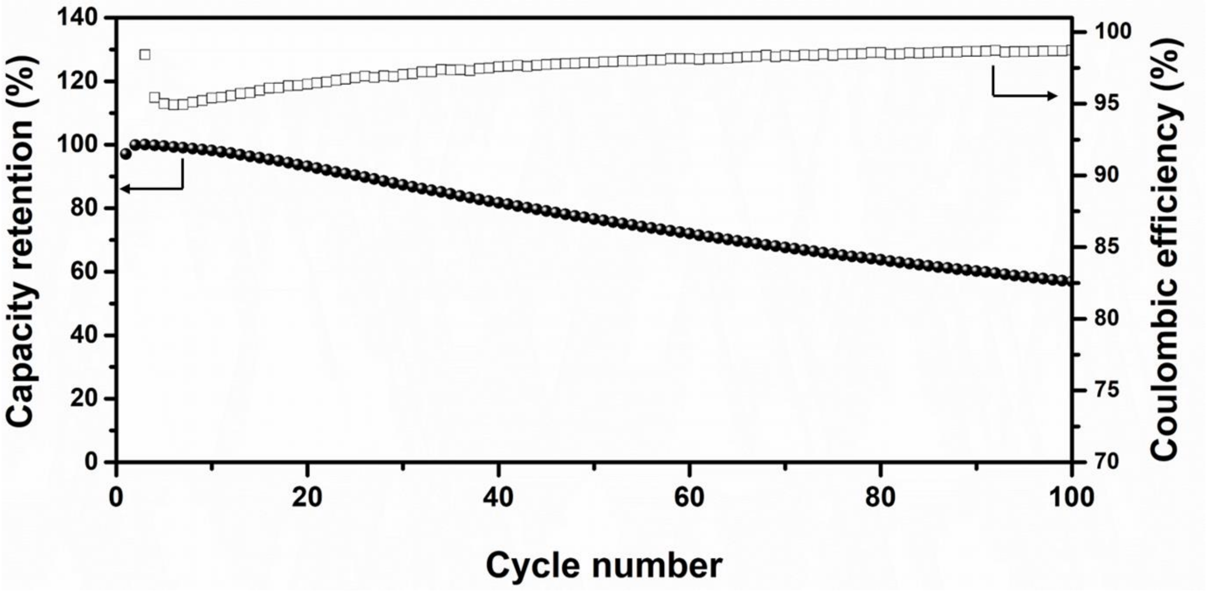

Supplementary Figure 21. Cycling performance of VOX electrode at 200 mA g^{-1} when paired with amalgamated zinc metal anode in the 3 m $ZnSO_{4(aq)}$ electrolyte.

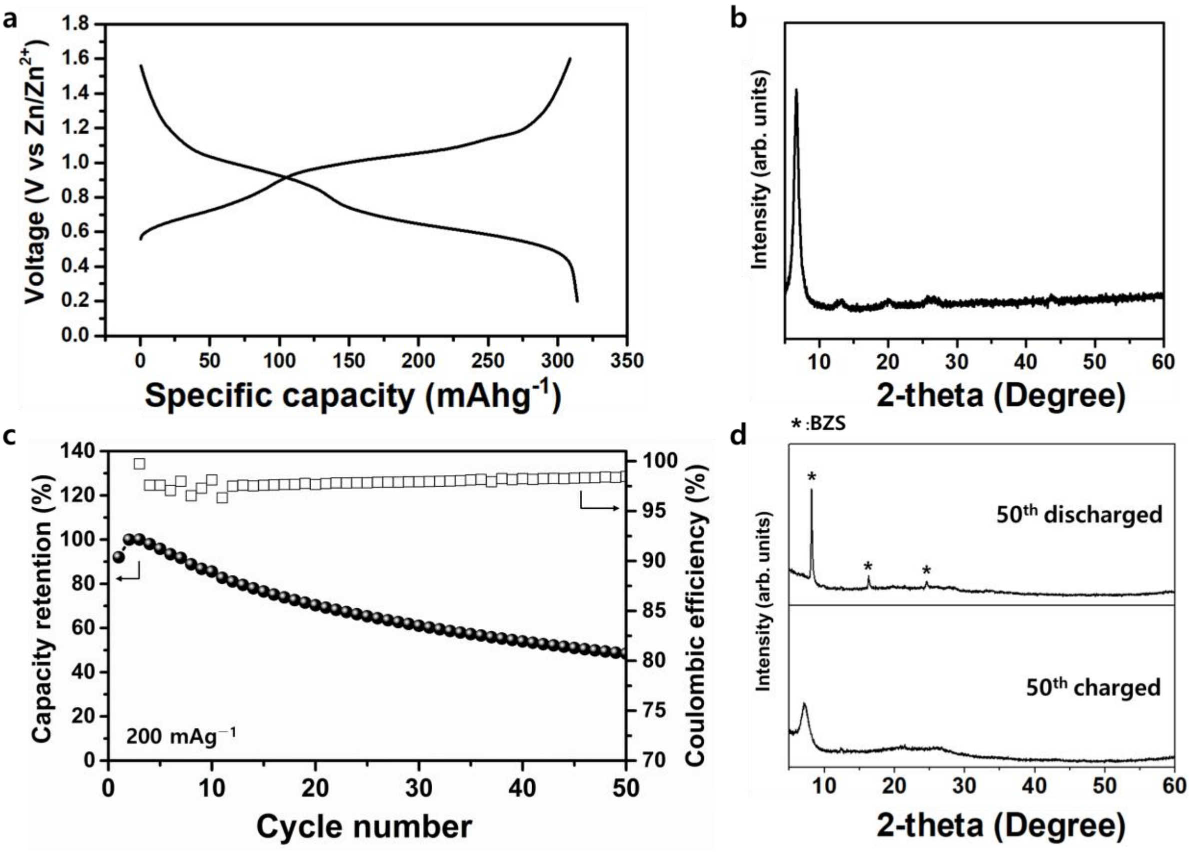

Supplementary Figure 22. Electrochemical behavior of VOX and its degradation analysis in 3 m $ZnSO_{4(aq)}$. **a**, Galvanostatic charge and discharge profiles of VOX electrode in its 1st cycle at 200 mA g^{-1} when the maximum capacity is achieved. **b**, XRD pattern of VOX electrode after 5 days of the dissolution test with an excess amount of electrolyte. **c**, Cycling performance of VOX electrode at 200 mA g^{-1}. **d**, Ex-situ XRD patterns of charged and discharged VOX electrodes after 50 cycles.

When 3 m $ZnSO_{4(aq)}$ was used as electrolyte, the maximum capacity of VOX electrode at 200 mA g^{-1} was 314 mAh g^{-1}, which is 18 % lower than that with the 3 m $Zn(OTf)_{2(aq)}$ electrolyte. Notably, the cell using 3 m $ZnSO_{4(aq)}$ retained only 48 % of the original capacity after 50 cycles, which is also inferior to that with the 3 m $Zn(OTf)_{2(aq)}$ electrolyte.
